# The splicing‐regulatory lncRNA NTRAS sustains vascular integrity

**DOI:** 10.15252/embr.202154157

**Published:** 2022-05-08

**Authors:** Youssef Fouani, Luisa Kirchhof, Laura Stanicek, Guillermo Luxán, Andreas W Heumüller, Andrea Knau, Ariane Fischer, Kavi Devraj, David John, Philipp Neumann, Albrecht Bindereif, Reinier A Boon, Stefan Liebner, Ilka Wittig, Carolin Mogler, Madina Karimova, Stefanie Dimmeler, Nicolas Jaé

**Affiliations:** ^1^ Institute of Cardiovascular Regeneration Centre of Molecular Medicine Goethe University Frankfurt Germany; ^2^ Faculty of Biological Sciences Goethe University Frankfurt Germany; ^3^ German Center of Cardiovascular Research (DZHK) Frankfurt Germany; ^4^ Department of Physiology Amsterdam Cardiovascular Sciences VU University Medical Center Amsterdam The Netherlands; ^5^ Institute of Neurology (Edinger Institute) University Hospital Goethe University Frankfurt Germany; ^6^ Institute of Biochemistry University of Giessen Giessen Germany; ^7^ Functional Proteomics Institute for Cardiovascular Physiology Goethe University Frankfurt Germany; ^8^ Institute of Pathology Technical University Munich Munich Germany; ^9^ Georg‐Speyer‐Haus Institute for Tumor Biology and Experimental Therapy Frankfurt Germany

**Keywords:** alternative splicing, long non‐coding RNA, tight junctions, vascular integrity, Cell Adhesion, Polarity & Cytoskeleton, RNA Biology, Vascular Biology & Angiogenesis

## Abstract

Vascular integrity is essential for organ homeostasis to prevent edema formation and infiltration of inflammatory cells. Long non‐coding RNAs (lncRNAs) are important regulators of gene expression and often expressed in a cell type‐specific manner. By screening for endothelial‐enriched lncRNAs, we identified the undescribed lncRNA NTRAS to control endothelial cell functions. Silencing of NTRAS induces endothelial cell dysfunction *in vitro* and increases vascular permeability and lethality in mice. Biochemical analysis revealed that NTRAS, through its CA‐dinucleotide repeat motif, sequesters the splicing regulator hnRNPL to control alternative splicing of tight junction protein 1 (TJP1; also named zona occludens 1, ZO‐1) pre‐mRNA. Deletion of the hnRNPL binding motif in mice (*Ntras* ^∆CA/∆CA^) significantly repressed TJP1 exon 20 usage, favoring expression of the TJP1α‐ isoform, which augments permeability of the endothelial monolayer. *Ntras* ^∆CA/∆CA^ mice further showed reduced retinal vessel growth and increased vascular permeability and myocarditis. In summary, this study demonstrates that NTRAS is an essential gatekeeper of vascular integrity.

## Graphical abstract

The dynamic regulation of the endothelial barrier is essential for molecule exchange and immune cell trafficking and, conversely, barrier failure is a hallmark of numerous disease processes. By sequestration of hnRNPL, the long non‐coding RNA NTRAS augments tight junction protein 1 (TJP1) exon 20 usage during splicing, eventually promoting endothelial resistance.

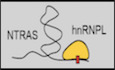

## Introduction

The vascular endothelium regulates the transit of plasma fluid, nutrients, waste products, and inflammatory cells between blood and organs. By interacting with its environment, the endothelial barrier is dynamically rearranged to maintain its selective permeability under physiological conditions. However, bacterial or viral infection‐triggered cytokine storms, as occurring during COVID‐19, can induce vascular leakage associated with edema formation and infiltration of inflammatory cells (Claesson‐Welsh, 2015; Libby & Lüscher, 2020; Teuwen *et al*, 2020). Barrier dysfunction additionally contributes to pathologies associated with increased angiogenesis, e.g., in tumors or during chronic inflammation (Nagy *et al*, 2008). To maintain vessel integrity, endothelial cells express adherens and tight junctions. Both types of junctions feature transmembrane adhesive proteins forming a pericellular zipper‐like structure and intracellular scaffold proteins, which mediate interactions with the actin cytoskeleton (Dejana, 2004). Tight junctions are multiprotein junctional complexes comprising the three major transmembrane proteins occludin, claudins, and junction adhesion molecules, which associate with different peripheral membrane proteins such as tight junction protein 1 (TJP1, also named ZO‐1). Encoded by the *TJP1* gene, this multidomain protein is located on the intracellular side of the plasma membrane to anchor the transmembrane junctional proteins to the actin component of the cytoskeleton (Campbell *et al*, 2017). Vascular barrier dysfunction is induced by various agonists such as histamine, thrombin, or VEGF, which augment intracellular calcium levels and induce post‐transcriptional signaling cascades (Wu *et al*, 1999; Bakker *et al*, 2002; Bogatcheva *et al*, 2002). Recent studies provide evidence that endothelial barrier function can also be controlled by RNA‐based mechanisms (Jaé & Dimmeler, 2020). Particularly, lncRNAs, which were initially mainly considered to regulate gene expression by interfering with imprinting, chromatin remodeling, and transcriptional regulation (Wang & Chang, 2011), were recently shown to directly interact with interfilament and other cytoskeletal‐associated proteins to control adherent junctions in endothelial cells (Lyu *et al*, 2019; Stanicek *et al*, 2020).

In this study, we determined the function of the novel endothelial‐enriched lncRNA NTRAS (non‐coding transcript regulating alternative splicing) in the vasculature, which controls alternative splicing of the critical tight junction protein TJP1 via an hnRNP‐dependent mechanism.

## Results

### NTRAS is essential for normal endothelial cell function and vital *in vivo*

**Figure EV1:**
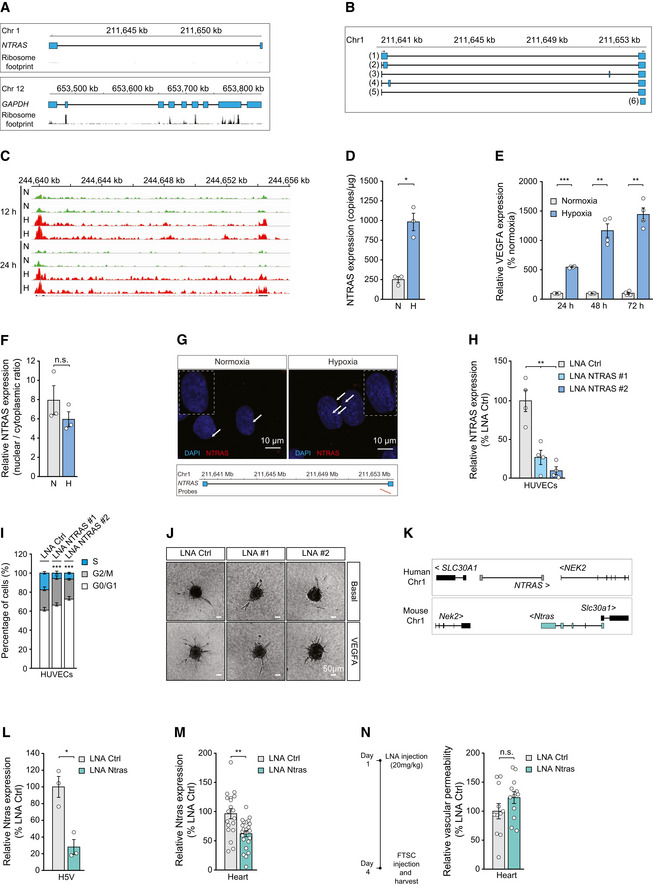
NTRAS is essential for normal endothelial cell function Illustration of the human *NTRAS* and *GAPDH* loci. Displayed are UCSC genome browser snapshots of ribosome profiling GWIPs‐viz ribose tracks.Schematic representation of NTRAS transcript variants identified by RT–PCR and 5’ RACE–PCR in HUVECs (*n* = 1).IGV screen shot showing NTRAS expression levels in HUVECs under normoxic (N) and hypoxic (H; 0.2% O_2_ for 12 h or 24 h) conditions (*n* = 2 independent biological replicates).Digital PCR‐based analysis of NTRAS copy numbers per µg of total RNA in HUVECs under normoxic (N) and hypoxic (H) conditions (*n* = 3 independent biological replicates).RT–qPCR‐based analysis of VEGFA expression, controlling for hypoxia (*n* = 4 independent biological replicates).Subcellular localization of NTRAS in normoxic (N) and hypoxic (H) HUVECs, assayed by cellular fractionation and RT–qPCR (*n* = 3 independent biological replicates).RNAscope‐based detection of NTRAS in HUVECs under normoxic and hypoxic conditions (*n* = 1). Binding sites of the probes are indicated.Validation of NTRAS silencing in HUVECs by RT–qPCR, comparing two different LNAs (*n* = 4 independent biological replicates).Cell cycle analysis in control and NTRAS‐silenced HUVECs (*n* = 3 independent biological replicates).Representative images of *in vitro* sprouting comparing control and NTRAS‐silenced HUVECs under basal conditions and VEGFA stimulation (*n* = 3–10 independent biological replicates). Scale bars are 50 µm.Illustration of the human and murine *NTRAS* loci (GRCh38.p13: RP11‐354k1.1; GRCm38/mm10: 1700034H15).Validation of Ntras silencing in murine H5V cells by RT‐qPCR (*n* = 3 independent biological replicates).RT–qPCR‐based analysis of Ntras expression in hearts of control and Ntras‐silenced mice (*n* = 19–23 mice per group).FTSC‐based *in vivo* permeability assays, comparing heart homogenates from control and Ntras‐silenced mice. Data normalized to organ and body weight (*n* = 11–12 mice per group). Experimental outline on the left. Data information: In (D–F, H, I, L–N), data are represented as mean ± SEM. n.s.: non‐significant, *P < 0.05, **P < 0.01, ***P < 0.001. (D–F, H, I, L–N) two‐tailed unpaired *t*‐test. Source data are available online for this figure.

**Figure 1:**
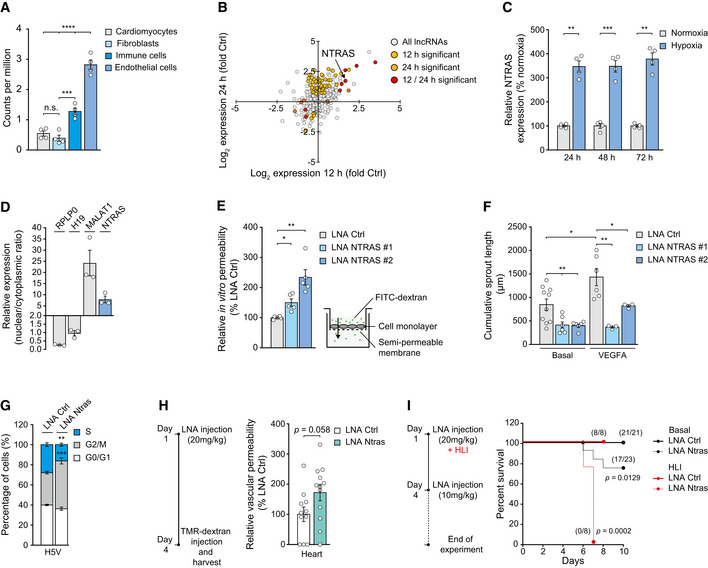
NTRAS is essential for normal endothelial cell function and vital *in vivo* Murine Ntras enrichment in endothelial cells versus other heart‐derived cell types (data acquired from GSE95755; Quaife‐Ryan *et al*, 2017) (*n* = 4 mice).Changes in lncRNA expression from HUVECs exposed to hypoxia (0.2% O_2_ for 12 h or 24 h), determined by ribo‐minus RNA deep sequencing (data acquired from GSE107033; Neumann *et al*, 2018); NTRAS is highlighted (*n* = 2 independent biological replicates).Validation of hypoxia‐induced NTRAS expression at the indicated time points by RT–qPCR (*n* = 4 independent biological replicates).Subcellular localization of NTRAS and control transcripts, assayed by nuclear‐cytoplasmic fractionation of HUVECs and RT–qPCR (*n* = 3 independent biological replicates).FITC‐dextran‐based *in vitro* permeability comparing control and NTRAS‐silenced HUVECs (*n* = 5 independent biological replicates).Cumulative *in vitro* sprout lengths of control and NTRAS‐silenced HUVECs under basal conditions and VEGFA stimulation (*n* = 3–10 independent biological replicates).Cell cycle analysis in Ntras‐silenced H5V cells (*n* = 3 independent biological replicates).TMR‐dextran‐based assessment of vascular permeability *in vivo*, comparing heart homogenates from control and Ntras‐silenced mice. Data normalized to organ and body weight (*n* = 11–12 mice per group). Experimental outline on the left.Survival of control and Ntras‐silenced mice under basal conditions (black, *n* = 21–23 mice per group) and in hind limb ischemia (HLI; red, *n* = 8 mice per group). Number of survivors is indicated; experimental outline on the left. Data information: In (A, C–H) data are represented as mean ± SEM. n.s.: non‐significant, *P < 0.05, **P < 0.01, ***P < 0.001, ****P < 0.0001. (A) One‐way ANOVA, (C–E, H) two‐tailed unpaired *t*‐test, (G) two‐way ANOVA, and (I) Mantel‐Cox‐test. Source data are available online for this figure.

In the present study, we dissect the role of the non‐protein coding, endothelial‐enriched transcript NTRAS (Figs EV1A and 1A) in maintaining vascular barrier function and integrity. We found the so far undescribed lncRNA NTRAS (RP11‐354k1.1) to be expressed in at least six transcripts variants in human umbilical vein endothelial cells (HUVECs) (Fig EV1B) and to be significantly and steadily induced by hypoxia (Figs 1B and C, and EV1C–E). Subcellular fractionation revealed a predominant nuclear localization of NTRAS (Fig 1D) which was maintained during hypoxia (Fig EV1F). *In situ* hybridization using exon as well as intron‐targeting probes confirmed the induction by hypoxia and the nuclear localization (Fig EV1G). Next, we asked whether NTRAS controls endothelial cell functions. Indeed, silencing of NTRAS by LNAs (Fig EV1H) reduced cell cycle progression (Fig EV1I), diminished endothelial barrier function (Fig 1E), and impaired basal and VEGF‐induced *in vitro* sprouting (Figs 1F and EV1J). Besides, NTRAS shows locus conservation in mice (Ntras, 1700034H15Rik), being flanked by *Nek2* and *Slc30a1* (Fig EV1K). This conservation further extends to functional conservation, as Ntras silencing in immortalized murine cardiac endothelial cells (H5V; Fig EV1L) halted cell cycle progression through S‐phase (Fig 1G). Moreover, *in vivo* silencing of Ntras by LNAs (Fig EV1M) increased vascular permeability as demonstrated by the increased tissue accumulation of fluorescently labeled TMR‐dextran or FTSC 4 days post knockdown (Figs 1H and EV1N). To investigate the angiogenic function of Ntras *in vivo*, hindlimb ischemia (HLI) was performed on control and Ntras‐silenced mice. Strikingly, all HLI‐challenged mice treated with Ntras‐targeting LNAs died 6–7 days post‐surgery, whereas all LNA control treated animals survived (Fig 1I, HLI). Interestingly, Ntras silencing alone already significantly reduced survival, evidenced by a ~ 25% reduction in survival rate (Fig 1I, basal). Collectively, these data demonstrate that the nuclear lncRNA NTRAS is essential for endothelial cell functions *in vitro* and intervening with its expression *in vivo* disrupts vascular integrity and impairs survival.

### NTRAS operates as splicing‐regulatory lncRNA

**Figure 2:**
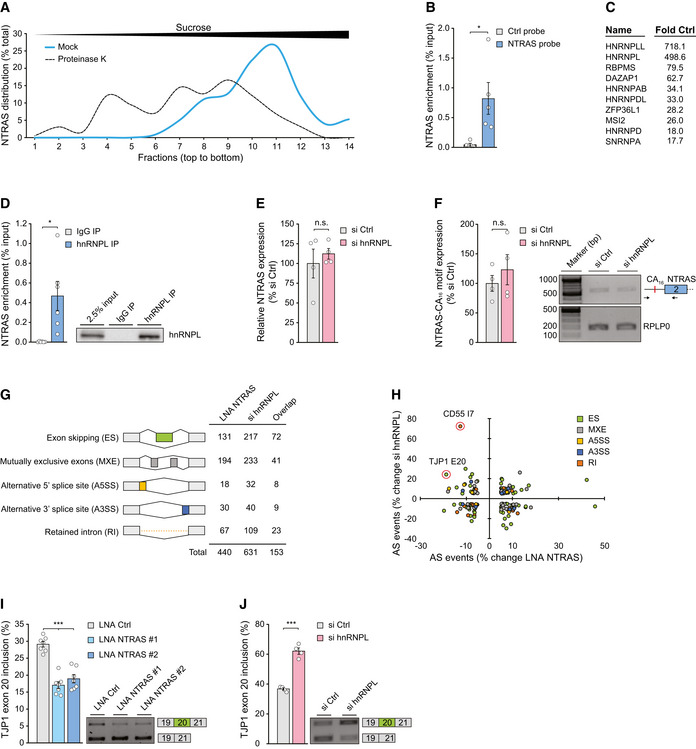
NTRAS operates as splicing‐regulatory lncRNA Separation of mock and proteinase K‐treated HeLa nuclear extracts by sucrose density gradient ultracentrifugation followed by NTRAS detection using RT–qPCR. Fractions 1 and 14 represent top and bottom of the gradient, respectively (*n* = 1).Enrichment of NTRAS by antisense affinity selection from HeLa cell lysate followed by RT–qPCR, comparing a control and an NTRAS‐specific probe (*n* = 5 independent biological replicates).Top ten enriched proteins from NTRAS affinity selections, identified by mass spectrometry (*n* = 5 independent biological replicates).RT–qPCR‐based validation of NTRAS–hnRNPL interaction by anti‐hnRNPL RIPs from HUVEC nuclear fractions (*n* = 6 independent biological replicates). Representative western blot on the right.RT–qPCR analysis of NTRAS expression in HUVECs upon silencing of hnRNPL (*n* = 4 independent biological replicates).RPLP0‐normalized NTRAS‐CA_16_ motif expression after hnRNPL silencing determined by RT‐PCR (*n* = 4 independent biological replicates).RNA deep sequencing‐based assignment of alternative splicing events in NTRAS‐ or hnRNPL‐silenced HUVECs, using rMATS software. Displayed are changes > 5% (*n* = 2 independent biological replicates).NTRAS and hnRNPL co‐regulated alternative splicing (AS) events. Data points with an FDR < 0.05 are circled (*n* = 2 independent biological replicates).RT–PCR‐based analysis of TJP1 exon 20 inclusion upon silencing of NTRAS in HUVECs (*n* = 7 independent biological replicates). Representative agarose gels on the right.RT–PCR‐based analysis of TJP1 exon 20 inclusion upon silencing of hnRNPL in HUVECs (*n* = 4 independent biological replicates). Representative agarose gels on the right. Data information: In (B, D–F, I, J), data are represented as mean ± SEM. n.s.: non‐significant, *P < 0.05, ***P < 0.001. (B–F, I, J) two‐tailed unpaired *t*‐test. Source data are available online for this figure.

**Figure EV2:**
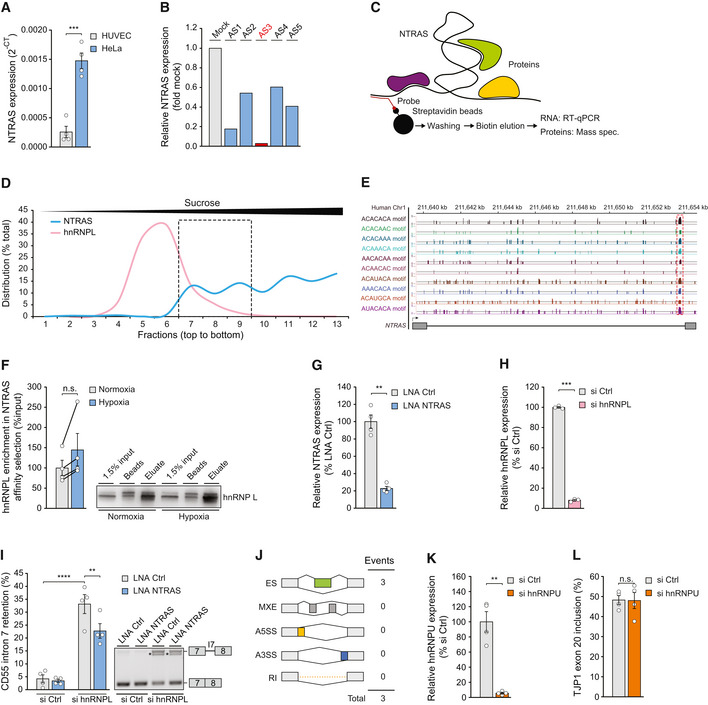
NTRAS operates as splicing‐regulatory lncRNA Relative expression of NTRAS in HUVECs and HeLa cells, determined by RT–qPCR (*n* = 4 independent biological replicates).RT–qPCR‐based identification of accessible regions within NTRAS using RNase H‐mediated cleavage of RNA–DNA heteroduplexes (DNA antisense oligonucleotides AS1 to AS5) in HUVEC cell lysate (*n* = 1). The oligonucleotide used for probe design is highlighted in red.Scheme illustrating the affinity selection of endogenous NTRAS–protein complexes for RNA and protein analysis.Sucrose density gradient ultracentrifugation showing the distribution of NTRAS and hnRNPL (protein). The dashed box indicates the fractions with the greatest overlap of both factors (*n* = 1).Illustration of the human NTRAS locus. Displayed is *NTRAS* (GRCh38.p13; RP11‐354k1.1) and RBPmap‐predicted hnRNPL binding motifs, described elsewhere (Smith *et al*, 2013).Western blot‐based validation of NTRAS–hnRNPL interaction following antisense affinity selection of NTRAS in nuclear extracts from normoxic and hypoxic HUVECs (*n* = 4 independent biological replicates).Expression of NTRAS in control and NTRAS‐silenced HUVECs used for RNA sequencing (*n* = 4 independent biological replicates).hnRNPL mRNA levels in control and hnRNPL‐silenced HUVECs used for RNA sequencing (*n* = 3 independent biological replicates).RT–PCR‐based analysis of CD55 intron 7 retention following hnRNPL/NTRAS double knockdown in HUVECs (*n* = 4 independent biological replicates). Representative agarose gel on the right.rMATs‐based analysis of alternative splicing events upon silencing of lncRNA lncflow2 in HUVECs (*n* = 4 independent biological replicates) ES: Exon skipping, MXE: Mutually exclusive exons, A5SS: Alternative 5’ splice site, A3SS: Alternative 3’ splice site, RI: Retained intron.Validation of hnRNPU silencing in HUVECs by RT–qPCR (*n* = 4 independent biological replicates).RT–PCR‐based analysis of TJP1 exon 20 inclusion in hnRNPU‐silenced HUVECs (*n* = 4 independent biological replicates). Data information: In (A, F–I, K, L), data are represented as mean ± SEM. n.s.: non‐significant, **P < 0.01, ***P < 0.001. (A, F–H, K, and L) two‐tailed unpaired *t*‐test, and (I) one‐way ANOVA. Source data are available online for this figure.

Next, we sought to characterize NTRAS mechanistically. To this end, we fractionated nuclear extracts by density gradient ultracentrifugation and uncovered NTRAS signals to shift toward lighter fractions upon proteinase K treatment, indicative of RNA–protein interactions (Figs 2A and EV2A). Subsequently, we purified these protein binding partners by antisense affinity selection. For this purpose, we first determined accessible regions within NTRAS using RNase H‐based cleavage of RNA–DNA heteroduplexes, followed by RT–qPCR (Fig EV2B). The most accessible sequence (AS3) was then used to design a 2′O‐Me‐RNA antisense probe carrying a 3′‐desthiobiotin‐TEG group for streptavidin selection of endogenous NTRAS–protein complexes (Fig EV2C). Recovered RNA fractions were analyzed for NTRAS enrichment (Fig 2B), and protein fractions were subjected to mass spectrometry, unraveling interactions with mainly splicing factors, in particular hnRNPL and hnRNPLL (heterogeneous nuclear ribonucleoprotein L and L‐like) (Fig 2C and Dataset EV1). Given that hnRNPL is a highly expressed protein (Beck *et al*, 2011) whereas NTRAS is rather a low abundant lncRNA, we questioned the stoichiometry of both factors. To this end, we deployed density gradient ultracentrifugation (Fig EV2D) revealing that the majority of hnRNPL (~79%) is not bound to NTRAS. However, a major fraction of NTRAS co‐sediments with hnRNPL, supporting the supposed interaction of both factors. This result is in line with the circumstance that hnRNPL is engaged in a multitude of different RNA‐binding processes, whereas the association with NTRAS might be involved in fine‐tuning a specific subset of hnRNPL‐mediated processes. In addition, *in silico* analysis of the NTRAS sequence revealed several CA‐rich hnRNPL binding motifs and strikingly a prominent *bona fide* hnRNPL binding site in the form of a CA_16_ repeat sequence proximal to the 3’ splice site of the predominantly retained intron 2 (Fig EV2E). Therefore, it might be reasonably assumed that the presence of multiple hnRNPL binding motifs within NTRAS will compensate for the unfavorable stoichiometry between both factors. Finally, RNA immunoprecipitation (Fig 2D) and RNA affinity selection followed by western blotting (Fig EV2F) unequivocally validated the interaction between NTRAS and hnRNPL. Furthermore, such interaction was enhanced under hypoxia‐mediated NTRAS upregulation, corroborating the aforementioned data (Fig EV2F). In summary, our results suggest that NTRAS exists as a constituent of an hnRNPL‐containing ribonucleoprotein complex in the nucleus.

Since hnRNPL is a well‐established splicing factor (Rothrock *et al*, 2005; Hung *et al*, 2008; Geuens *et al*, 2016), and we could exclude the contribution of hnRNPL to NTRAS expression and processing (Fig 2E and F), we speculated that NTRAS might also be involved in splicing regulation. To analyze this, we used RNA deep sequencing to evaluate whether NTRAS modulates splicing in HUVECs. Notably, NTRAS or hnRNPL silencing (Fig EV2G and H) regulated 440 and 631 individual splicing events, respectively (Fig 2G). Among those, exon skipping and mutually exclusive exons represented the most abundant modes of alternative splicing to be altered (Fig 2G) and both factors individually displayed no preference acting as splicing repressor or activator (Dataset EV2). Interestingly, 153 NTRAS‐controlled splicing events were also under hnRNPL surveillance (Fig 2G overlap, and Dataset EV3), and individual silencing of NTRAS or hnRNPL resulted case‐specifically in opposing or similar splicing outcomes (Fig 2H and Dataset EV3). When applying more stringent criteria (FDR ≤ 0.05), two co‐regulated events became evident: tight junction protein 1 (TJP1) exon 20 usage and CD55 intron 7 retention (Fig 2H). In both cases, NTRAS silencing was found to counteract hnRNPL silencing, favoring skipping of TJP1 exon 20 (Fig 2I and J, Appendix Fig S1A) and splicing of CD55 intron 7 (Fig EV2I). Of note, silencing of an unrelated control lncRNA and hnRNPU, a heterogeneous nuclear ribonucleoprotein not associated with NTRAS, failed to regulate TJP1 exon 20 inclusion rates (Fig EV2J–L).

In conclusion, our results show that NTRAS and hnRNPL co‐regulate alternative splicing of a shared set of target pre‐mRNAs in a case‐specific manner. Furthermore, we identify TJP1 and CD55 as major target transcripts of this splicing‐regulatory tandem.

### NTRAS sustains endothelial barrier function by promoting TJP1α+ expression

**Figure EV3:**
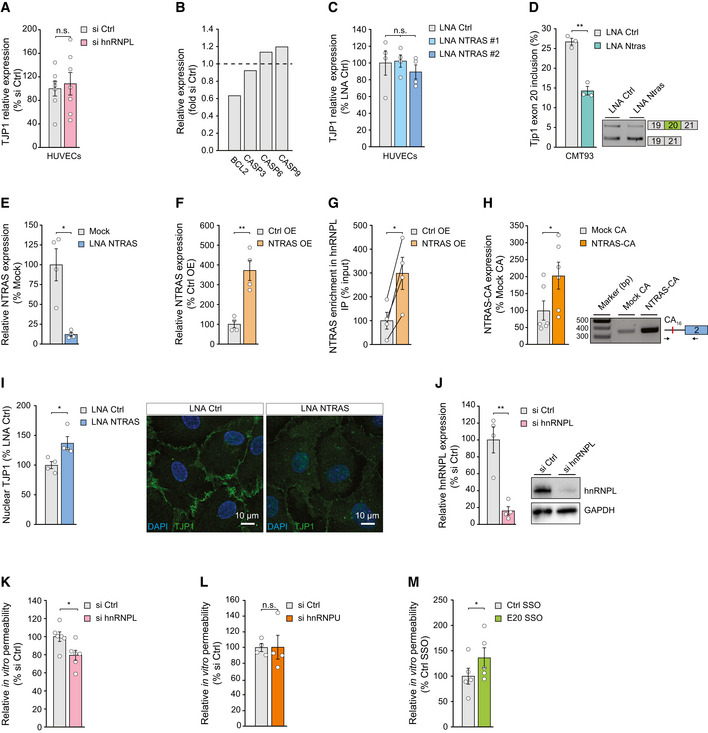
NTRAS controls TJP1 splicing and endothelial barrier function RT–qPCR‐based analysis of TJP1 mRNA expression in hnRNPL‐silenced HUVECs (*n* = 7 independent biological replicates).Relative expression of BCL2, CASP3, CASP6, and CASP9 mRNA in hnRNPL‐silenced HUVECs (*n* = 2 independent biological replicates).RT–qPCR‐based analysis of TJP1 mRNA expression in NTRAS‐silenced HUVECs (*n* = 4 independent biological replicates).RT–PCR‐based analysis of Tjp1 exon 20 inclusion in control and Ntras‐silenced murine CMT93 epithelial cells (*n* = 3 independent biological replicates).RT–qPCR‐based validation of RNase H‐mediated NTRAS degradation in HeLa nuclear extracts used for *in vitro* splicing (*n* = 4 independent biological replicates).RT–qPCR‐based validation of NTRAS overexpression (*n* = 4 independent biological replicates).Co‐precipitation of NTRAS in anti‐hnRNPL RIPs, using nuclear lysates from control and NTRAS‐overexpressing cells (*n* = 4 independent biological replicates).RT–PCR‐based validation of NTRAS‐CA_16_ motif overexpression (*n* = 6 independent biological replicates). Representative agarose gel on the right.Quantification of nuclear TJP1, comparing control and NTRAS‐silenced HUVECs (*n* = 4 independent biological replicates). Representative micrographs are shown. Scale bars are 10 µm.Analysis of hnRNPL knockdown by western blot 72 h post‐transfection of control or hnRNPL‐targeting siRNAs (*n* = 4 independent biological replicates). Representative western blots on the right.
*In vitro* permeability assays using FITC‐dextran, comparing control and hnRNPL‐silenced HUVECs (*n* = 6 independent biological replicates).
*In vitro* permeability assays using FITC‐dextran, comparing control and hnRNPU‐silenced HUVECs (*n* = 4 independent biological replicates).
*In vitro* permeability assays using FITC‐dextran, comparing control SSO‐ and E20 SSO‐transfected HUVECs (*n* = 5 independent biological replicates). Data information: In (A, C–M) data are represented as mean ± SEM. n.s.: non‐significant, *P < 0.05, **P < 0.01. (A–I) two‐tailed unpaired *t*‐test. Source data are available online for this figure.

As a scaffold protein, TJP1 mediates the interaction between integral tight junction proteins, e.g., occludins and claudins, and the actin cytoskeleton and is important for tight junction function (Fanning *et al*, 1998; Fanning & Anderson, 2009). On protein level, TJP1 is expressed as TJP1α+ or TJP1α−, discriminated by the presence or absence of an 80 amino acid alpha domain, respectively. Importantly, this alpha domain is encoded by the alternative exon 20 (Balda & Anderson, 1993). Considering both the augmented permeability upon NTRAS silencing and the changes in TJP1 exon 20 selection during splicing, we assumed that NTRAS, together with hnRNPL, controls endothelial barrier function through TJP1 exon 20. In this context, a recently reported regulation of TJP1 total expression levels (and apoptosis‐related proteins) by hnRNPL in epithelial cells (Lv *et al*, 2017) could not be observed for endothelial cells (Fig EV3A and B). Likewise, NTRAS silencing in endothelial cells did not influence TJP1 total mRNA levels (Fig EV3C). However, exon 20 splicing regulation by NTRAS was also evident in the epithelium (Fig EV3D).

**Figure 3:**
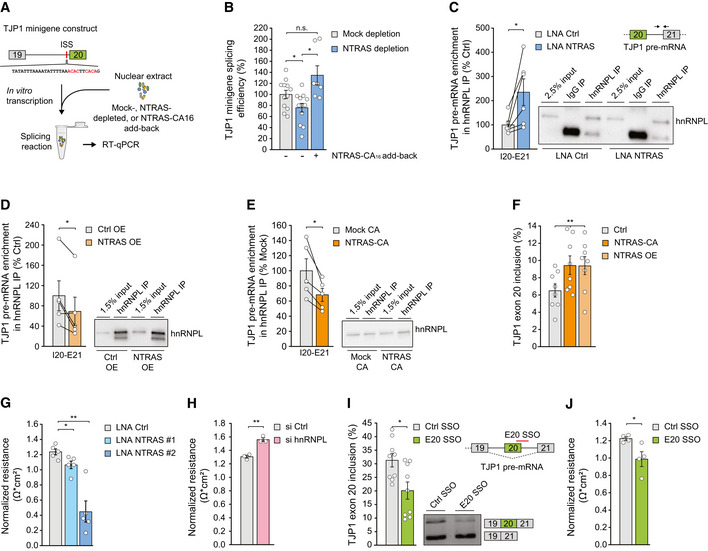
NTRAS controls TJP1 splicing and endothelial barrier function Scheme depicting the *in vitro* splicing of a TJP1 exon 19–exon 20 splice substrate featuring an intronic splicing silencer (ISS). hnRNPL binding sites are highlighted in red.
*In vitro* splicing efficiency of the TJP1 splice substrate, comparing mock, NTRAS‐depleted, and NTRAS‐CA_16_ motif add‐back conditions (*n* = 7–12 independent biological replicates).Co‐precipitation of TJP1 pre‐mRNA in anti‐hnRNPL RIPs, using nuclear lysates from control and NTRAS‐silenced HUVECs (*n* = 6 independent biological replicates).Co‐precipitation of TJP1 pre‐mRNA in anti‐hnRNPL RIPs, using nuclear lysates from control and NTRAS‐overexpressing cells (*n* = 5 independent biological replicates). Representative western blot on the right.Co‐precipitation of TJP1 pre‐mRNA in anti‐hnRNPL RIPs, using nuclear lysates from control and NTRAS‐CA_16_ motif overexpressing cells (*n* = 5 independent biological replicates). Representative western blot on the right.RT–PCR‐based analysis of TJP1 exon 20 inclusion upon NTRAS overexpression and overexpression of the NTRAS‐CA_16_ motif (*n* = 8 independent biological replicates).Endothelial resistance of NTRAS‐silenced HUVECs (*n* = 3–5 independent biological replicates), analyzed by electrical cell‐substrate impedance sensing (ECIS).Endothelial resistance of hnRNPL‐silenced HUVECs (*n* = 3 independent biological replicates), analyzed by ECIS.Analysis of TJP1 exon 20 inclusion by RT–PCR upon transfection of HUVECs with a control SSO or an SSO masking the exon 20–intron 20 boundary (E20 SSO) (*n* = 9 independent biological replicates). Representative agarose gel on the right. Schematic outline at the top right.Endothelial resistance of control SSO‐ or E20 SSO‐transfected HUVECs (*n* = 4 independent biological replicates), analyzed by ECIS. Data information: In (B–J), data are represented as mean ± SEM. n.s.: non‐significant, *P < 0.05, **P < 0.01. (B, G–J) two‐tailed unpaired *t*‐test, (C–E) two‐tailed paired *t*‐test, and (F) one‐way ANOVA. Source data are available online for this figure.

We therefore sought to mechanistically analyze in depth the role of the NTRAS‐hnRNPL axis in regulating TJP1 exon 20 usage. First, we assessed the *in vitro* splicing efficiency of a TJP1 minigene construct upon NTRAS depletion in splicing competent nuclear extract. Since the *in vitro* transcription of an exon 19‐20‐21 TJP1 minigene proved to be inefficient, we deployed a previously described construct, comprising the constitutive exon 19, intron 19 (which contains the hnRNPL binding motifs), and the alternative exon 20 (Fig 3A) (Heiner *et al*, 2010). RNase H‐mediated NTRAS degradation in nuclear extracts prior to splicing (Fig EV3E) significantly diminished the splicing efficiency of the TJP1 exon 19–20 minigene (Fig 3B). Strikingly, this effect could be rescued by the addition of an *in vitro* transcribed NTRAS fragment, harboring the CA_16_ dinucleotide repeat, prior to splicing (Fig 3B). Second, we observed an enhanced co‐precipitation of TJP1 pre‐mRNA in anti‐hnRNPL RNA immunoprecipitation assays (RIPs) upon NTRAS silencing (Fig 3C). Vice versa, overexpression of full‐length NTRAS augmented its association with hnRNPL (Fig EV3F and G) and significantly reduced TJP1 pre‐mRNA co‐precipitation (Fig 3D). Third, overexpression of the CA_16_ repeat alone resembled these results by reducing the co‐precipitation of TJP1 pre‐mRNA in anti‐hnRNPL RIPs (Figs EV3H and 3E). Finally, and in line with the previous results, overexpression of full‐length NTRAS or the CA_16_ repeat significantly drives TJP1 splicing toward exon 20 inclusion (Fig 3F).

Taken together, these results demonstrate that TJP1 exon 20 is partially regulated by NTARS through sequestration of hnRNPL and, in this context, underlines the central functional role of the NTRAS‐CA_16_ repeat: hnRNPL binds to TJP1 pre‐mRNA and acts as a repressor of exon 20 leading to exon skipping; NTRAS with its hnRNPL binding sites competes with TJP1 pre‐mRNA for hnRNPL binding thereby augmenting exon 20 inclusion.

Next, we addressed the contribution of NTRAS, hnRNPL, and TJP1 exon 20 to endothelial barrier function by electrical cell‐substrate impedance sensing (ECIS) in HUVECs. NTRAS silencing significantly decreased endothelial resistance (Fig 3G) and altered the subcellular distribution of TJP1 (Fig EV3I). In contrast, silencing of hnRNPL (Fig EV3J) specifically augmented barrier function (Figs 3H and EV3K), whereas silencing of the non‐specific splicing factor hnRNPU had no effect (Fig EV3L). Furthermore, to clarify whether endothelial resistance is directly determined by TJP1α+ expression, we used a splice‐switching oligonucleotide (E20 SSO) to interfere with TJP1 exon 20 recognition (Fig 3I), this way mimicking NTRAS silencing directly at the level of splicing. Strikingly, SSO‐mediated exon 20 skipping phenocopied NTRAS silencing, resulting in significantly impaired barrier function (Figs 3J and EV3M). Collectively, these data connect the splicing‐regulatory tandem NTRAS‐hnRNPL to endothelial barrier function through TJP1 exon 20 selection. Furthermore, silencing of NTRAS and hnRNPL, as well as TJP1 exon 20 manipulation using splice‐switching oligonucleotides, highlights the importance of the exon 20‐encoded alpha domain in maintaining endothelial junctions’ integrity.

### Ntras sustains vascular integrity *in vivo*

**Figure 4:**
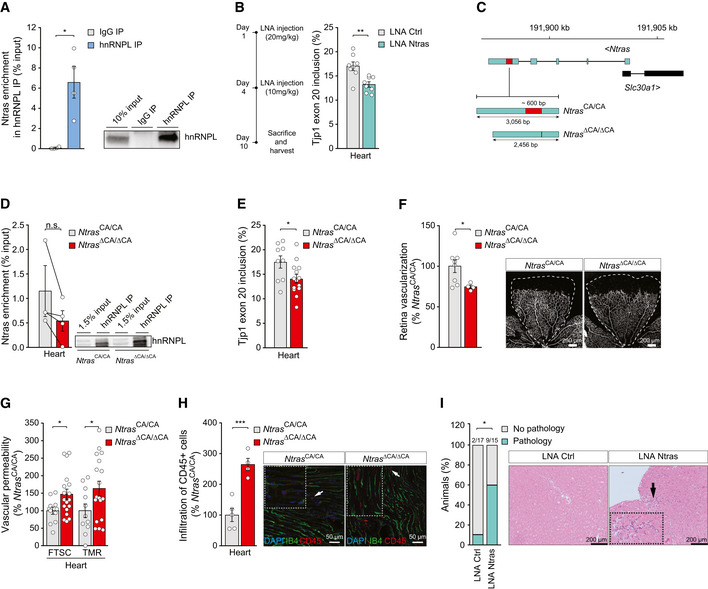
Ntras sustains vascular integrity *in vivo* Validation of Ntras–hnRNPL interaction by anti‐hnRNPL RIPs from H5V lysates, followed by RT–qPCR (*n* = 4 independent biological replicates). Representative western blot on the right.RT–PCR‐based analysis of murine Tjp1 exon 20 inclusion in cardiac tissue from control and Ntras‐silenced mice (*n* = 8 mice per group). Experimental outline on the left.Illustration of the murine *Ntras* locus. Displayed are *Ntras* and *Slc30a1* (GRCm38/mm10). The red box highlights the deleted genomic region (~600 bp) in *Ntras* ^∆CA/∆CA^ mice, comprising the hnRNPL binding motif (~130 bp) and flanking sequences (~440 bp).Co‐precipitation of Ntras in anti‐hnRNPL RIPs using whole heart lysates from *Ntras* ^CA/CA^ and *Ntras* ^∆CA/∆CA^ mice (*n* = 3–4 mice per group). Representative western blot on the right.RT–PCR‐based analysis of TJP1 exon 20 inclusion in hearts from *Ntras* ^CA/CA^ and *Ntras* ^∆CA/∆CA^ mice (*n* = 9–14 mice per group).Retinal angiogenesis assessed in P7 *Ntras* ^CA/CA^ and *Ntras* ^∆CA/∆CA^ pups by immunostaining of isolectin B4. Vascularized areas were normalized to total retinal area (*n* = 4–8 mice per group). Representative micrographs are shown. Scale bars are 200 µm.FTSC and TMR‐dextran *in vivo* permeability assays, comparing homogenates of hearts from *Ntras* ^CA/CA^ and *Ntras* ^∆CA/∆CA^ mice. Data normalized to organ and body weight (*n* = 11–18 mice per group).Quantification of CD45+ cell (red) infiltration into cardiac tissue from *Ntras* ^CA/CA^ and *Ntras* ^∆CA/∆CA^ mice normalized to DAPI. Isolectin B4 (green) was used to label endothelial cells (*n* = 4–5 mice per group). Representative micrographs are shown. Scale bars are 50 µm. Insets and arrows indicate sites of CD45+ cell infiltration.Percentage of control and Ntras‐silenced mice showing cardiac pathologies (*n* = 15–17 mice per group). Prevalence is indicated above the bars. H&E micrographs of murine heart sections from control and Ntras‐silenced mice are shown. Insets and arrows indicate sites of lymphocytic infiltration. Scale bars are 100 µm. Data information: In (A, B, D–H), data are represented as mean ± SEM. n.s.: non‐significant, *P < 0.05, **P < 0.01, ***P < 0.001. (A) two‐tailed paired *t*‐test, (B, D–H) two‐tailed unpaired *t*‐test, and (I) Chi‐square test. Source data are available online for this figure.

**Figure EV4:**
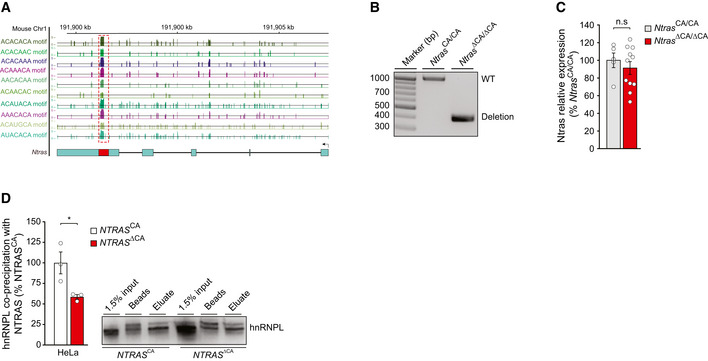
Characterization of the Ntras CA‐repeat *in vivo* Illustration of the murine *Ntras* locus. Displayed is *Ntras* (GRCm38/mm10; 1700034H15) and RBPmap‐predicted hnRNPL binding motifs, described elsewhere (Smith *et al*, 2013).Genotyping results confirming the genomic deletion of the hnRNPL binding motif in *Ntras* ^∆CA/∆CA^ mice.RT–qPCR‐based analysis of Ntras expression in *Ntras* ^CA/CA^ and *Ntras* ^∆CA/∆CA^ mice (*n* = 5–11 mice per group).Western blot‐based analysis of the NTRAS–hnRNPL interaction following antisense affinity selection of NTRAS in nuclear fractions from *NTRAS* ^CA^ controls and *NTRAS* ^∆CA^ HeLa cells (*n* = 3 independent biological replicates). Representative western blot on the right. Data information: In (C, D), data are represented as mean ± SEM. n.s.: non‐significant, *P < 0.05. (C, D) two‐tailed unpaired *t*‐test. Source data are available online for this figure.

Based on our mechanistic findings *in vitro* and the observation that Ntras‐silenced mice exhibited cardiac hyperpermeability and inflammation, we hypothesized that Ntras also controls vascular integrity *in vivo* through TJP1 exon 20 splicing. Indeed, RIPs revealed an interaction between Ntras and murine hnRNPL (Fig 4A). Similar to its human homologue, murine Ntras also harbors an extended hnRNPL CA_65_ binding motif (Fig EV4A). Consistently, LNA‐mediated silencing of Ntras *in vivo* reduced Tjp1 exon 20 inclusion (Fig 4B), indicative of a mechanistic conservation.

Next, to corroborate that the aforementioned findings result from dysfunctional Ntras transcripts, we generated C57BL/6J‐*Ntras* ^ΔCA/ΔCA^ mice (hereafter referred to as *Ntras* ^ΔCA/ΔCA^), lacking the CA_65_ repeat (Figs 4C and EV4A and B). Importantly, the total expression level of Ntras in these mice was not influenced by the deletion (Fig EV4C). In line with results obtained by deleting the NTRAS‐CA_16_ repeat in cell culture (Fig EV4D), deletion of the corresponding nucleotides *in vivo* tended to reduce the interaction between Ntras and hnRNPL (Fig 4D). Strikingly, Tjp1 exon 20 usage was significantly diminished in those *Ntras* ^ΔCA/ΔCA^ mice (Fig 4E), this way confirming the crucial role Ntras plays in fine‐tuning Tjp1 splicing and isoform expression through sequestering hnRNPL.

Physiologically and in accordance with the angiogenesis defects observed *in vitro*, *Ntras* ^ΔCA/ΔCA^ mice showed a delayed retinal vessel growth (Fig 4F) and exhibited a significant increase in vascular permeability (Fig 4G), resembling the vascular hyperpermeability upon LNA‐mediated Ntras silencing in mouse (compare Fig 1H). Finally, a histopathological examination of heart tissue from *Ntras* ^ΔCA/ΔCA^ mice revealed an increased inflammatory response, evidenced by infiltration of CD45‐positive cells (Fig 4H), consistent with a higher prevalence of cardiac pathologies associated with increased infiltration of lymphocytic cells in Ntras‐silenced mice (Fig 4I). In conclusion, these results highlight the pivotal role of Ntras and its conserved hnRNPL binding motif in maintaining vascular integrity and restricting inflammation.

## Discussion

**Figure 5:**
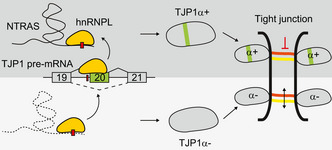
NTRAS controls vascular permeability by regulating TJP1 splicing Model depicting the molecular and physiological barrier function of NTRAS. By sequestering hnRNPL, NTRAS promotes TJP1 exon 20 inclusion and expression of TJP1α+, this way maintaining endothelial barrier function. In the absence of NTRAS, hnRNPL represses exon 20, thereby favoring TJP1α− expression and hyperpermeability. TJP1 alternative exon 20 and the alpha domain are highlighted in green; hnRNPL binding motifs are depicted as red boxes.

Our results unveil the conserved lncRNA NTRAS as novel gatekeeper of vascular integrity and inflammation, preserving the equilibrium between TJP1α+ and α− isoforms by regulating alternative splicing. Using biochemical purification strategies, we show that NTRAS competes with TJP1 pre‐mRNA for binding of the splicing factor hnRNPL to promote the inclusion of TJP1 alternative exon 20 (Fig 5). TJP1 is known to be expressed in two major isoforms (α+ and α−), defined by the presence or absence of an 80 amino acid alpha domain (Kurihara *et al*, 1992; Willott *et al*, 1992; Balda & Anderson, 1993) encoded by the alternative exon 20. The physiological implications of TJP1 isoform expression with respect to barrier function, however, are conflicting and not conclusively characterized: Early on, TJP1α− was suggested to be expressed in the context of open intercellular spaces, e.g., in the slit diaphragms of rat kidneys (Kurihara *et al*, 1992). Another study assigned TJP1 isoform expression to tight junction dynamics with TJP1α− being expressed in fine‐tuning of alternative splicing structurally more flexible junctions (Balda & Anderson, 1993). Our study reveals that NTRAS silencing and interference with TJP1 exon 20 recognition promotes TJP1α− isoform expression, leading to increased endothelial permeability (Fig 5). In turn, promotion of TJP1α+ expression, e.g., by silencing of hnRNPL, showed a barrier protective effect. In line with these results, mice silenced for Ntras showed a significantly reduced survival and signs of impaired vascular permeability. Moreover, specific deletion of the Ntras‐CA motif confirmed the importance of the hnRNPL binding sequence within Ntras, which is responsible for TJP1 exon 20 recognition and vascular integrity *in vivo*. Together, these results strongly support the idea that intercellular barrier function is co‐determined by TJP1 isoform expression and establish NTRAS as endothelial barrier protector. However, it remains to be fully defined how these insights come into effect during *de novo* barrier formation or dynamic remodeling. In this context, our results show that postnatal retina vascularization in *Ntras* ^ΔCA/ΔCA^ mice and *in vitro* sprouting of NTRAS‐silenced human endothelial cells are significantly impaired and accompanied by augmented TJP1 exon 20 skipping. Of note, these results are in line with a recent study demonstrating that TJP1α+ expression is diminished during epithelial‐to‐mesenchymal transition (Kim *et al*, 2019), a highly dynamic process characterized by the loss of intercellular junctions (Lamouille *et al*, 2014). In the current work, we specifically delineate the role of NTRAS‐hnRNPL in TJP1 splicing and endothelial integrity; nevertheless, our genome‐wide splicing analysis of co‐regulated events suggest further reaching impact. In this context, we were able to validate numerous additional co‐regulated splicing events (Appendix Fig S1B–F), arguing for the involvement of NTRAS‐hnRNPL in a splicing‐regulatory network that extends beyond the regulation of TJP1 exon 20. In addition, our data imply that both NTRAS and hnRNPL can act case‐specifically as splicing activators or repressors. This duality is established for RNA‐binding proteins, including hnRNPL (Motta‐Mena *et al*, 2010), and is generally linked to their position‐dependent binding within the regulated pre‐mRNA (Lee & Rio, 2015). The respective mechanisms for lncRNAs, however, are not well understood. While lncRNAs can in the simplest case regulate alternative splicing by hijacking splicing factors, they also can form RNA–RNA duplexes with their target pre‐mRNAs, thus masking splice sites or recruiting splicing factors. The latter is, for example, described for the lncRNA BC200, which regulates Bcl‐x pre‐mRNA splicing through base pairing and recruitment of hnRNPA2/B1 (Singh *et al*, 2016). While we provide evidence that NTRAS competes with TJP1 pre‐mRNA for hnRNPL binding, it is intriguing to speculate that other synergistically regulated splice substrates might rely on an lncRNA‐based recruitment of hnRNPL, eventually contributing to the observed endothelial phenotype.

Taken together, a sound understanding of the molecular mechanisms and pathways of vascular permeability is key for the development of targeted therapeutic strategies. In this context, lncRNAs might prove as potent, novel targets since first studies just begun to indicate their contribution to intercellular permeability (Lyu *et al*, 2019; Stanicek *et al*, 2020). With the characterization of NTRAS, its splicing‐regulatory network, and the discovery of TJP1 exon 20‐dependent barrier properties, we integrate the scrupulously regulated process of alternative splicing as central determinant of vascular integrity, opening up novel possibilities to intervene with barrier dysfunction.

## Materials and Methods

### Oligonucleotides

Primers, DNA oligonucleotides, and siRNAs were purchased from Sigma‐Aldrich, LNAs from Qiagen, and splice‐switching oligonucleotides and 2′O‐Me‐RNA probes from Integrated DNA Technologies. Stabilized one‐piece sgRNAs were synthetized by Synthego. All sequences are listed in Dataset EV4.

### Cell culture

Pooled human umbilical vein endothelial cells (HUVECs; Lonza) were cultured in endothelial basal medium, supplemented with EGM SingleQuots (Lonza) and 10% FCS (Invitrogen). HeLa cells and immortalized murine heart endothelial cells (H5V) were cultured in DMEM (Gibco) with 10% FCS and 1% penicillin/streptomycin. All cells were cultured at 37°C, 5% CO_2_ and tested negative for mycoplasma. Hypoxia was induced by incubation at 0.2% O_2_ for the indicated time points. For silencing of gene expression, cells were transfected with LNAs (50 nM) or siRNAs (67 nM), using Lipofectamine RNAiMax (Life Technologies) according to the manufacturer’s instructions.

### Deep sequencing and bioinformatics

For the identification of hypoxia‐regulated lncRNAs in endothelial cells, library preparation, RNA sequencing, and mapping was performed as described elsewhere (Neumann *et al*, 2018). The data set can be accessed via the Gene Expression Omnibus database with the identifier GSE107033. For the analysis of alternative splicing, ribosomal RNA‐depleted RNA from controls, or NTRAS‐ or hnRNPL‐silenced HUVECs was fragmented, primed with random hexamers, and processed according to the protocol of the Illumina TrueSeq RNA Library Prep Kit v2. Sequencing reads were mapped to the human reference genome GRCh38 by STAR (version 2.5.2.), and alternative splicing events were identified using rMats software (version 3.2.5) with default parameters. The enrichment of hnRNPL‐ and PTBP1‐binding motifs was done by using RBPmap (Paz *et al*, 2014), while putative NTRAS–RNA interactions were assessed using IntaRNA (Mann *et al*, 2017).

### RNA isolation, RT–(q)PCR, and RACE

Total RNA from cells and tissues was isolated and DNase digested using miRNeasy Kits (Qiagen), according to the manufacturer’s instructions. Homogenization of tissue samples in Qiazol was done using a FastPrep 24 Homogenizer (3 × 20 s strokes with 5 min pause), followed by centrifugation (15,000 rpm, 15 min, 4°C). For RT–qPCR, cDNA was synthesized from 500 ng RNA, using random hexamers and M‐MLV reverse transcriptase (Thermo Fisher). qRT–PCR reactions were performed with Fast SYBR Green on StepOnePlus real‐time PCR systems (Thermo Fisher). RPLP0 amplification was used for data normalization, and relative expression levels were calculated by 2^−ΔCt^. For semiquantitative RT–PCR, cDNA was synthesized from 250 ng RNA and amplified by Platinum DNA Taq Polymerase (Thermo Fisher). 5′ RACE–PCR reactions were performed using the 5′/3′ RACE kit, 2^nd^ generation (Roche) according to the manufacturer’s procedure. PCR products were identified by Sanger sequencing, visualized by MIDORI Green agarose gel electrophoresis and quantified by Image Lab software (version 5.2.1.).

### RNAScope

RNA *in situ* hybridization was performed with the RNAScope Multiplex Fluorescent Assay v2 (Advanced Cell Diagnostics, Bio‐Techne) according to manufacturer’s instructions. Briefly, formaldehyde‐fixed samples were dehydrated through immersions in serial ethanol dilutions for 5 min each, before air drying at RT for 5 min. After creating a hydrophobic barrier, samples were rehydrated and permeabilized for 10 min using 0.1% Tween 20–PBS solution. Samples were washed twice with PBS and incubated in hydrogen peroxide for 10 min at RT, then washed twice with PBS. Subsequently, samples were treated with protease III for 10 min at RT and washed twice with PBS. Hybridization with NTRAS probes and amplification were performed according to the instructions. For signal detection, samples were incubated with TSA Cyanine 5 (Perkin Elmer). Nuclei were counterstained with DAPI. Samples were mounted with Fluoromount‐G (Thermo Fisher). Images were taken using a Leica Stellaris 8 confocal microscope at 63× magnification.

### BrdU cell proliferation assays

Cell proliferation assays were performed using the BrdU Flow Kit (BD Bioscience) according to manufacturer’s protocol. Transfected HUVECs or H5V cells were incubated with BrdU (0.1 mM) for 3 h at 37°C. Next, cells were washed thoroughly with PBS, Cytofix/Cytoperm buffer, Perm/Wash buffer, and Cytoperm Plus buffer. Subsequently, cells were incubated in DNase I solution for 1 h at 37°C, washed with Perm/Wash buffer, and further incubated with V450 mouse anti‐BrdU antibodies (clone 3D4) for 20 min at RT. Finally, 7‐AAD was added for 10 min at RT and cells were analyzed, using a FACS Canto II device and FACSDiva software (BD Bioscience).

### *In vitro* sprouting assays

Spheroid sprouting assays were carried out as described elsewhere (Neumann *et al*, 2018). Briefly, HUVECs were transfected with LNAs for 24 h, trypsinized, and added to a mixture of medium and methylcellulose (80%:20%) in U bottom‐96‐well plates to allow for the formation of spheroids. After 24 h at 37°C, spheroids were collected, embedded in a rat‐tail collagen type‐I gel (BD Biosciences), and further incubated under basal conditions or VEGFA stimulation (50 ng/ml). Finally, gels were fixed with 10% formaldehyde and images were taken, using an Axio Observer Z1.0 microscope (Zeiss) at 10× magnification. The cumulative sprout length of each spheroid was measured by using the Zeiss AxioVision digital imaging software (version 4.6). Ten spheroids were analyzed per group per replicate.

### *In vitro* permeability assays

Transfected HUVECs were trypsinized and 1.2 × 10^5^ cells were seeded on fibronectin‐coated 24‐well thincerts (pore diameter 1 µm; Greiner Bio‐One). Next, cells were cultured for 24 h with 250 µl and 850 µl of EBM medium in the upper and lower chamber, respectively. After washing twice with PBS, 250 µl of FITC‐dextran (Sigma) in Opti‐MEM I (1 mg/ml; gibco) was added to the upper chamber, while 850 µl Opti‐MEM was added to the lower chamber. Following incubation for 1 h at 37°C, aliquots from the lower chamber were taken and fluorescence (λex = 493 nm, λem = 518 nm) was measured using a GloMax‐Multi+ Detection System (Promega).

### Animal experiments

All animal experiments were carried out in accordance with the principles of laboratory animal care as well as according to the German national laws. The studies have been approved by the local ethic committee (Regierungspräsidium Darmstadt, Hessen). For LNA‐mediated Ntras silencing, 12‐week‐old C57BL/6J mice were injected intraperitoneally with LNAs targeting Ntras or control LNAs (20 mg/kg on day 1 and 10 mg/kg on day 4).

### *In vivo* permeability assays

12–14‐week‐old male or female, *Ntras* ^CA/CA^, *Ntras* ^ΔCA/ΔCA^, and LNA‐treated mice were intravenously injected with 100 µl of a 1:1 mixture of 2 mM tetramethylrhodamine‐dextran (TMR‐dextran; 3 kDa) and 5 mM fluorescein‐5‐thiosemicarbazide (FTSC; 0.4 kDa). The tracers were allowed to circulate for 5 min before mice were sacrificed and perfused with PBS. Hearts were collected and homogenized in PBS using a FastPrep 24 Homogenizer (3 × 20 s strokes with 5 min pause). Supernatants were cleared (15,000 rpm, 15 min, 4°C) and fluorescence (λex = 555 nm, λem = 585 nm for TMR‐dextran and λex = 490 nm, λem = 520 nm for FTSC) was measured, using a Synergy HT reader and Gen5 software (BioTek).

### Hindlimb ischemia

For HLI experiments, 12‐week‐old female C57BL/6J mice were injected intraperitoneally with LNAs, as mentioned above. Animals received buprenorphine (0.1 mg/kg) as an analgesic 30 min prior to and 12 h post‐surgery. Additionally, ampicillin (0.1 mg/g) was administered via the drinking water. The procedure was performed under general anesthesia using isoflurane delivered by mask. Following skin disinfection with povidone‐iodine, the superficial femoral artery was exposed proximally to the external iliac artery by an incision in the right hind leg, extending ~1.0 cm from the inguinal ligament to the distal side. Next, the external iliac, the profound femoral, and the superficial femoral arteries were ligated, followed by the removal of the proximal section of the superficial femoral artery. The incision was subsequently sutured. Analgesia was continued with carprofen (5 mg/kg) for up to 3 days.

### Cellular fractionation

Human umbilical vein endothelial cells were fractionated using NE‐PER Extraction Kits (Thermo Fisher) according to the manufacturer’s instructions. Briefly, normoxic or hypoxic (0.2% O_2_ for 24 h) cells were washed, pelleted, lysed in ice‐cold CER I buffer, and incubated for 10 min on ice. Then, ice‐cold CER II buffer was added and samples were incubated for additional 1 min on ice. Following centrifugation (16,000 *g*, 5 min, 4°C), cytoplasmic supernatants were taken for RNA preparation. Nuclei were resuspended and lysed in nucleic NER buffer for 40 min on ice. Lysates were cleared (16,000 *g*, 10 min, 4°C) and RNA was prepared from nucleic supernatants.

### Sucrose density gradient ultracentrifugation

RNA–protein complexes from HeLa nuclear extracts (Ipracell) were separated by sucrose density ultracentrifugation (15–55% sucrose; 1.059–1.258 g/cm^3^) for 2.5 h at 200,620 *g* and 4°C using an MLS‐50 rotor (Beckman Coulter). Prior to centrifugation, 100 µl HeLa nuclear extracts were incubated for 30 min at 37°C with 0.5 mg proteinase K and 400 U RiboLock. For mock treatment, proteinase K was substituted by RNase‐free H_2_O. Next, reaction volumes were adjusted to 900 µl with 5% sucrose solution and 800 µl were loaded on top of the gradient. After centrifugation, 14 fractions were taken for RNA isolation and cDNA synthesis. NTRAS levels were determined by qPCR and the equation 2^−Ct^.

### RNA accessibility assays

Human umbilical vein endothelial cells were lysed in lysis buffer (50 mM Tris–HCl pH 8, 150 mM NaCl, 0.5% NP‐40, protease inhibitor) and cleared lysates were adjusted to 1.1 ml with 110 µl 10× RNase H‐Buffer (NEB), NaCl_2_ (60 mM final), and H_2_O. Next, 100 µl reactions were mixed with 100 pmol DNA oligonucleotides and incubated for 2 h at 4°C. Thereafter, 2.5 U RNase H (NEB) were added and reactions were kept for 20 min at 37°C. Finally, RNA was isolated for RT–qPCR.

### RNA antisense affinity selection and mass spectrometry

HeLa cells were lysed in lysis buffer (50 mM Tris–HCl pH 8, 50 mM NaCl, 0.5% NP‐40, 80 U RiboLock, protease inhibitor) and volumes were adjusted to 1 ml with the same buffer lacking NP‐40. For selection of RNP complexes, lysates were pre‐cleared for 2 h at 4°C using blocked streptavidin C1 beads (yeast tRNA and glycogen, both 0.2 mg/ml). Next, lysates were incubated with 100 pmol desthiobiotin‐labeled 2′O‐Me‐RNA oligonucleotides overnight at 4°C. Complexes were captured using 25 µl blocked beads for 1 h at 37°C. Beads were washed thoroughly with washing buffer (50 mM Tris–HCl pH 8, 50 mM NaCl, 0.05% NP‐40) and eluted with biotin (50 µM) at RT. Eluates were analyzed by RT–qPCR, western blotting, and mass spectrometry using a Q Exactive Plus mass spectrometer. The proteomics data together with a detailed method description will be deposited to the ProteomeXchange Consortium via the PRIDE (Perez‐Riverol *et al*, 2019) partner repository. For extended data analysis, the data set was loaded to Perseus 1.5.2.6 (Tyanova *et al*, 2016), cleaned from reverse identifications, only identified by side and common contaminants. Identifications were filtered for 4 valid values in at least one group (*n* = 5). Missing values were replaced from normal distribution. Student’s *t*‐test was used to identify significantly enriched proteins.

### RNA immunoprecipitation (RIP)

Cells were lysed using NE‐PER Extraction Kits (Thermo Fisher) according to the manufacturer’s instructions. Next, 50 µl Protein G Dynabeads (Thermo Fisher) were coupled with 12 µg anti‐hnRNPL antibody (ab6106; Abcam) or a serotype control (CS200621; Millipore) overnight at 4°C in binding buffer (50 mM Tris–HCl pH 8, 50 mM NaCl). HnRNPL was immunoprecipitated from nuclear fractions overnight at 4°C. After washing the beads three times with washing buffer (50 mM Tris–HCl pH 8, 50 mM NaCl, 0.05% NP‐40), RNA was recovered and subjected to RT‐qPCR. Aliquots of input and precipitated material were analyzed by western blotting.

### Western blot

Cells were lysed in 1 × RIPA buffer (Thermo Fisher) supplemented with protease inhibitor cocktail (Roche Diagnostics). Protein concentrations were determined by Bradford assays (Bio‐Rad). Forty‐five micrograms of proteins was separated by SDS–PAGE and electroblotted to PVDF membranes (Millipore). Membranes were blocked in 5% BSA in TBS‐T for 1 h at RT. Antibodies detecting hnRNPL (ab6106, Abcam; 1:1,000) and GAPDH (14C10, Cell Signaling Technology; 1:1,000) were diluted in blocking solution and incubated overnight. For detection, membranes were incubated with HRP‐conjugated anti‐mouse or anti‐rabbit secondary antibodies (GE‐Healthcare) for 1 h at RT. Blots were developed using Immobilon western chemiluminescent HRP substrate (Millipore) and imaged using a ChemiDoc Touch Imaging System (Bio‐Rad).

### *In vitro* transcription and splicing

The TJP1 minigene constructs (kindly provided by A. Bindereif, JLU Giessen) are explicitly described elsewhere (Heiner *et al*, 2010). For *in vitro* transcription, 1 µg of PCR‐generated DNA template was incubated in a 25 µl reaction mix (1× transcription buffer, 10 mM DTT, 0.5 mM ATP, 0.5 mM UTP, 0.5 mM CTP, 0.1 mM GTP, 0.4 U Ribo m7G cap analog, 10 U RiboLock, 10 U T7 RNA polymerase) for 1 h at 37°C. Following DNase digestion for 30 min at 37°C, pre‐mRNA was phenolized and ethanol precipitated. For splicing analysis, 10 ng *in vitro* transcribed pre‐mRNA were incubated in 120 µl HeLa nuclear extract (Ipracell) supplemented with ATP (0.1 mM), MgCl_2_ (0.6 mM), creatine phosphate (3.8 mM), poly‐vinyl alcohol (0.5%), and RiboLock for 2 h at 37°C. Next, splicing reactions were proteinase K digested and RNA was phenolized, ethanol precipitated, and used for RT‐PCR.

### NTRAS activation by CRISPR‐SAM

1 × 10^6^ HeLa cells were seeded in high‐glucose DMEM medium and transduced the next day with ~2.4 × 10^3^ viral particles of CRISPR helper construct 1 (SAMVP64BSTV; Sigma) and ~18.75 × 10^3^ particles of helper construct 2 (SAMMS2HYGV; Sigma) in medium containing 8 µg/ml polybrene (Sigma). Seventy‐two hours post‐transduction, positively transduced cells were double‐selected and maintained in medium containing 5.5 µg/ml blasticidin (Sigma) and 9 µg/ml hygromycin (Sigma) for 10 days. Subsequently, 2 × 10^5^ helper 1^+^/helper 2^+^ cells were transduced with ~2.2 × 10^4^ viral particles of an NTRAS targeting‐gRNA construct (LV07; Sigma) in medium supplemented with 8 µg/ml polybrene (Sigma). Positively transduced cells were selected using 750 µg/ml zeocin (Thermo Fisher) for 10 days. NTRAS levels were determined by qPCR.

### NTRAS‐CA motif overexpression

The NTRAS‐CA motif genomic region was amplified from HUVEC gDNA, followed by cloning into pcDNA3.1+ (Addgene). Next, 1 × 10^6^ cells were seeded in 10‐cm dishes and transfected at 80% confluency with 5 µg of the respective construct using 50 µl GeneJuice (Millipore). Finally, overexpression of the NTRAS‐CA motif was validated by RT‐PCR.

### CRISPR Cas9‐mediated NTRAS‐CA motif deletion

Guide RNAs targeting the NTRAS‐CA motif were designed (http://crispr.mit.edu/) and cloned in pLentiCRISPRv2‐GFP‐Puromycine, kindly provided by Dr. Madina Karimova. For virus production, these plasmids were co‐transfected with psPAX2 and pMD2.G in Lenti‐X 293T cells (Takara), using GeneJuice (Millipore) according to manufacturer’s protocol. Viruses were collected 24 h and 48 h post‐transfection. Mock pLentiCRISPRv2‐GFP‐Puromycine plasmids were used to produce control viruses. Next, 1 × 10^6^ HeLa cells were seeded in high‐glucose DMEM medium and transduced the next day with a pool of NTRAS‐CA motif‐targeting or control viruses in medium supplemented with 8 µg/ml polybrene (Sigma). Seventy‐two hours post‐transduction, cells were selected and maintained in medium containing 1 µg/ml puromycin (Invitrogen) for 14 days. The deletion of the CA motif was validated by genotyping.

### Electric cell‐substrate impedance sensing (ECIS)

Electrical cell‐substrate impedance sensing was performed to assess endothelial barrier function following transfection of HUVECs with LNAs, SSOs, or siRNAs. Twenty‐four hours following transfection, 4 × 10^4^ cells per well were seeded on gelatin‐coated 96W1E^+^ PET plates (Applied BioPhysics). The resistance of the monolayer (Rb) was measured for 48 h following seeding, using the ECIS instrument Zθ (Applied BioPhysics) set to an alternating current of 400 Hz. Data were analyzed using the ECIS software (version 1.2.123.).

### Generation of *Ntras* ^ΔCA/ ΔCA^ mice

Four‐ to six‐week‐old female C57BL/6J mice (Charles River Laboratories) were superovulated by intraperitoneal injection of 5 IU pregnant mare serum gonadotropin (ProSpec‐Tany TechnoGene), followed by injection of 5 IU human chorionic gonadotropin (ProSpec‐Tany TechnoGene) 48 h later. Superovulated females were mated 1:1 with 3–8‐month‐old C57BL/6J males to generate one‐cell fertilized zygotes at 0.5 days post‐coitum (dpc). The female animals were sacrificed at 0.5 dpc, oviducts were collected, and oocyte–cumulus complexes released and dissociated from cumulus cells by hyaluronidase (Sigma‐Aldrich) treatment at final concentration 300 µg/ml in M2 medium (Millipore). To weaken the zona pellucida, embryos underwent a 30–60 s treatment of acid Tyrode’s solution (Sigma‐Aldrich), followed by 5 washes with M2 medium. sgRNA–Cas9 complexes were assembled in Opti‐MEM at a final concentration of 8 µM, using a 1:1.5 ratio of NLS‐Cas9 protein (IDT) and sgRNA. Prior to electroporation, 20–40 zygotes were pooled and washed once with Opti‐MEM. Subsequently, groups of zygotes in 10 µl Opti‐MEM were combined with 10 µl sgRNA–Cas9 complexes and loaded into electroporation cuvettes. A standard square wave electroporation was performed using two pulses at 30 V for 3 ms, separated by a 100 ms interval. The next day, successfully developed two‐cell embryos were transferred in M2 medium into the oviducts of 0.5 dpc CD1 pseudopregnant females, with approximately 10 embryos per oviduct. Viable pups were subjected to genotyping analysis.

### Immunofluorescence and histology

For TJP1 immunofluorescence, 4 × 10^4^ cells were cultured on gelatin‐coated 8‐well µ‐Slides (ibidi). Cells were fixed in methanol for 15 min at RT, permeabilized in 0.1% Triton X‐100 in PBS, and blocked for 1 h (3% BSA, 5% donkey serum, PBS‐T). Next, cells were incubated overnight at 4°C with primary anti‐TJP1 antibodies (40‐2200, Thermo Fisher; 1 : 50), washed with PBS, and incubated with Alexa‐Fluor 488 secondary antibodies (Invitrogen, 1 : 200) in 5% BSA for 1 h at RT. Nuclei were counterstained with DAPI and cells were mounted with Fluoromount‐G (Thermo Fisher). Images were taken using a Leica TCS SP8 confocal microscope at 63× magnification. Images were analyzed using Volocity (version 6.5.). For histological analysis, harvested mouse hearts were fixed in 4% formaldehyde overnight at 4°C, embedded in paraffin and sectioned. For hematoxylin and eosin (H&E) staining, a standard procedure (Cardiff *et al*, 2014) for processing 2 μm sections was followed. For immunofluorescence, 50 µm OCT‐embedded heart sections were permeabilized in PBS‐T (0.1% Triton X‐100 in PBS) and blocked for 1 h (3% BSA, 5% donkey serum, PBS‐T). Next, sections were incubated overnight at 4°C with primary anti‐CD45 antibodies (30F‐11, BD Pharmingen; 1:100) and biotinylated isolectin B4 (B‐1205, Vector Laboratories; 1:50). Samples were then washed with PBS and incubated with Alexa‐Fluor 555‐conjugated secondary antibodies or Alexa‐Fluor 647‐conjugated streptavidin (Invitrogen, 1:200) in 5% BSA for 2 h at RT. Nuclei were stained with DAPI and sections were mounted with Fluoromount‐G (Thermo Fisher). Images were taken using a Leica TCS SP8 confocal microscope at 40× magnification. Images were analyzed using Volocity (version 6.5.).

### Mouse retinal angiogenesis model

Eyes from postnatal Ntras^ΔCA/ΔCA^ mice (P7) were harvested and fixed in 4% formaldehyde for 1 h at 4°C. Following washing with PBS, retinas were dissected, partially cut into four leaflets, and blocked for 1 h at RT in blocking solution (1% BSA, 2% goat serum, 0.5% Triton X‐100 in PBS). Next, retinas were incubated with biotinylated isolectin B4 (B‐1205, Vector Laboratories; 1:50) in diluted blocking solution (0.5% BSA, 1% goat serum, 0.25% Triton X‐100 in PBS) overnight at 4°C. After washing with 0.1% Triton X‐100 in PBS, retinas were incubated with Alexa‐Fluor 647 conjugated streptavidin (Invitrogen; 1:400) in PBS. Retinas were flat‐mounted using Fluoromount‐G (Thermo Fisher) and images were taken using a Leica TCS SP8 microscope at 10× magnification. Vascularized areas of all four leaflets per retina were measured and normalized to the overall surface area of the leaflet using Volocity (version 6.5.).

### Statistical analysis

Data are expressed as means ± SEM and were tested for outliers using the ROUT method. Shapiro–Wilk normality test was used for data normality assessment. Unless otherwise stated, statistical significance was assessed by two‐tailed paired *t*‐test, two‐tailed unpaired *t*‐test, or Mann–Whitney *U* test. Multiple comparisons were performed using one‐way or two‐way ANOVA using Tukey’s or Dunnett’s correction. Probability values of <0.05 were considered significant. *n* refers to the number of independent biological replicates.

## Author contributions

**Youssef Fouani:** Conceptualization; Data curation; Formal analysis; Validation; Investigation; Visualization; Methodology; Writing—original draft; Writing—review and editing. **Luisa Kirchhof:** Data curation; Formal analysis; Validation; Investigation; Methodology; Writing—review and editing. **Laura Stanicek:** Formal analysis; Investigation. **Guillermo Luxán:** Formal analysis; Investigation. **Andreas W Heumüller:** Formal analysis; Validation; Investigation; Writing—review and editing. **Andrea Knau:** Investigation. **Ariane Fischer:** Formal analysis; Validation; Investigation. **Kavi Devraj:** Investigation. **David John:** Data curation; Software; Formal analysis. **Philipp Neumann:** Validation; Investigation; Methodology. **Albrecht Bindereif:** Resources; Methodology; Writing—review and editing. **Reinier A Boon:** Resources; Writing—review and editing. **Stefan Liebner:** Resources; Methodology. **Ilka Wittig:** Data curation; Software; Formal analysis; Investigation; Visualization. **Carolin Mogler:** Resources; Data curation; Validation; Investigation; Methodology. **Madina Karimova:** Data curation; Formal analysis; Validation; Investigation; Methodology. **Stefanie Dimmeler:** Conceptualization; Data curation; Supervision; Funding acquisition; Methodology; Writing—original draft; Project administration; Writing—review and editing. **Nicolas Jaé:** Conceptualization; Data curation; Supervision; Funding acquisition; Investigation; Methodology; Writing—original draft; Project administration; Writing—review and editing.

In addition to the CRediT author contributions listed above, the contributions in detail are:

YF designed and performed experiments, analyzed data, and drafted the paper. LK, LS, AWH, AK, PN, and KD performed experiments. AF performed animal experiments DJ performed bioinformatics analysis. GL and CM performed and analyzed immunostainings. IW performed mass spectrometry. MK generated Ntras^ΔCA/ΔCA^ mice. AB, RAB, and SL provided conceptual input. SD and NJ supervised the project, designed experiments, analyzed data, and drafted the paper.

## Disclosure and competing interests statement

RAB, SD, and NJ applied for the following patent that is related to NTRAS: International PCT Patent Application No. PCT/EP2016/052246 "LONG NON‐CODING RNA FOR THE TREATMENT OF ENDOTHELIAL DYSFUNCTION". Filed 03 February 2016.

## Supporting information



Appendix

Expanded View Figures PDF

Dataset EV1

Dataset EV2

Dataset EV3

Dataset EV4

Source Data for Expanded View

Source Data for Figure 1

Source Data for Figure 2

Source Data for Figure 3

Source Data for Figure 4

## Data Availability

RNA deep sequencing data were deposited at the ArrayExpress database under the accession number E‐MTAB‐11311 (http://www.ebi.ac.uk/arrayexpress/experiments/E‐MTAB‐11311/). The proteomics data are uploaded to the ProteomeXchange Consortium and can be accessed via the accession number PXD030620 (https://www.ebi.ac.uk/pride/archive/projects/PXD030620).
