# Supplementary material for: The splicing‐regulatory lncRNA NTRAS sustains vascular integrity
Source: EMBO Rep. 2022 May 8;23(6):e54157. doi: 10.15252/embr.202154157 (PMC9171682; doi:10.15252/embr.202154157)
Supplement: Supplementary file 1 — Appendix [file EMBR-23-e54157-s006.pdf]

Appendix

- Appendix Figure S1

Appendix Figure S1

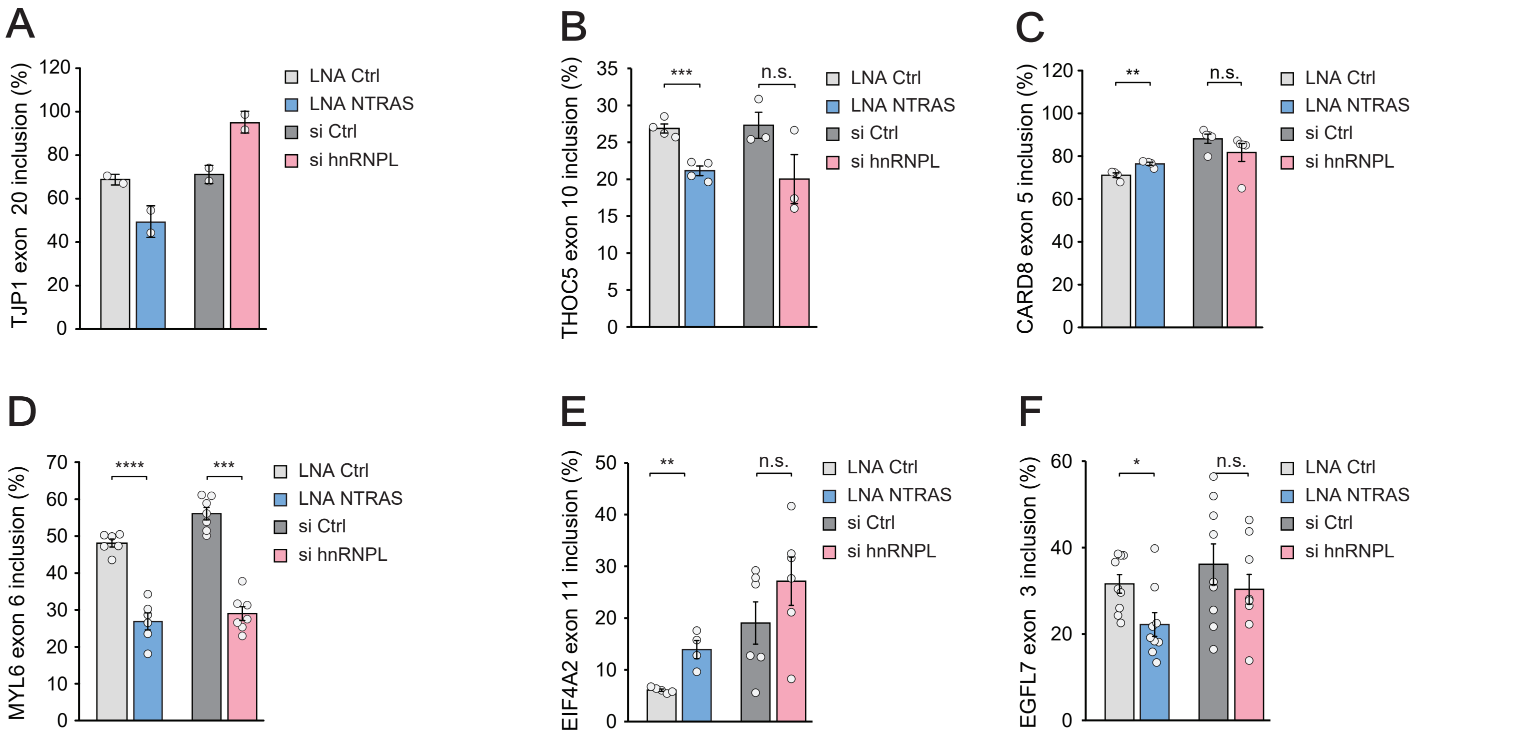

Appendix Figure S1 | NTRAS and hnRNPL co-regulated alternative splicing (AS) events.

(A) RNA deep sequencing-based analysis of TJP1 exon 20 inclusion upon silencing of NTRAS or hnRNPL in HUVECs (n = 2 independent biological replicates). Data refers to Fig 2H **(B)** RT-PCR-based analysis of THOC5 exon 10 (n = 3-4 independent biological replicates), **(C)** CARD8 exon 5 (n = 5-6 independent biological replicates), **(D)** MYL6 exon 6 (n = 6-7 independent biological replicates), **(E)** EIF4A2 exon 11 (n = 4-6 independent biological replicates), and **(F)** EGFL7 exon 3 (n = 9 independent biological replicates) inclusion in NTRAS- or hnRNPL-silenced HUVECs.

Data information: In (A-E) data are represented as mean ± S.E.M. n.s.: non-significant, *p < 0.05, **p < 0.01, ***p < 0.001, ****p < 0.0001.
