# Supplementary material for: The splicing‐regulatory lncRNA NTRAS sustains vascular integrity
Source: EMBO Rep. 2022 May 8;23(6):e54157. doi: 10.15252/embr.202154157 (PMC9171682; doi:10.15252/embr.202154157)
Supplement: Supplementary file 2 — Expanded View Figures PDF [file EMBR-23-e54157-s001.pdf]

Expanded View Figures

◀ **Figure EV1. NTRAS is essential for normal endothelial cell function.**

A Illustration of the human *NTRAS* and *GAPDH* loci. Displayed are UCSC genome browser snapshots of ribosome profiling GWIPs-viz ribose tracks.
B Schematic representation of NTRAS transcript variants identified by RT–PCR and 5' RACE–PCR in HUVECs (n = 1).
C IGV screen shot showing NTRAS expression levels in HUVECs under normoxic (N) and hypoxic (H; 0.2% O_2 for 12 h or 24 h) conditions (n = 2 independent biological replicates).
D Digital PCR-based analysis of NTRAS copy numbers per μg of total RNA in HUVECs under normoxic (N) and hypoxic (H) conditions (n = 3 independent biological replicates).
E RT–qPCR-based analysis of VEGFA expression, controlling for hypoxia (n = 4 independent biological replicates).
F Subcellular localization of NTRAS in normoxic (N) and hypoxic (H) HUVECs, assayed by cellular fractionation and RT–qPCR (n = 3 independent biological replicates).
G RNAscope-based detection of NTRAS in HUVECs under normoxic and hypoxic conditions (n = 1). Binding sites of the probes are indicated.
H Validation of NTRAS silencing in HUVECs by RT–qPCR, comparing two different LNAs (n = 4 independent biological replicates).
I Cell cycle analysis in control and NTRAS-silenced HUVECs (n = 3 independent biological replicates).
J Representative images of *in vitro* sprouting comparing control and NTRAS-silenced HUVECs under basal conditions and VEGFA stimulation (n = 3–10 independent biological replicates). Scale bars are 50 μm.
K Illustration of the human and murine *NTRAS* loci (GRCh38.p13: RP11-354k1.1; GRCm38/mm10: 1700034H15).
L Validation of Ntras silencing in murine H5V cells by RT-qPCR (n = 3 independent biological replicates).
M RT–qPCR-based analysis of Ntras expression in hearts of control and Ntras-silenced mice (n = 19–23 mice per group).
N FTSC-based *in vivo* permeability assays, comparing heart homogenates from control and Ntras-silenced mice. Data normalized to organ and body weight (n = 11–12 mice per group). Experimental outline on the left.

Data information: In (D–F, H, I, L–N), data are represented as mean ± SEM. n.s.: non-significant, *$P < 0.05$, **$P < 0.01$, ***$P < 0.001$. (D–F, H, I, L–N) two-tailed unpaired t-test.
Source data are available online for this figure.

 © 2022 The Authors

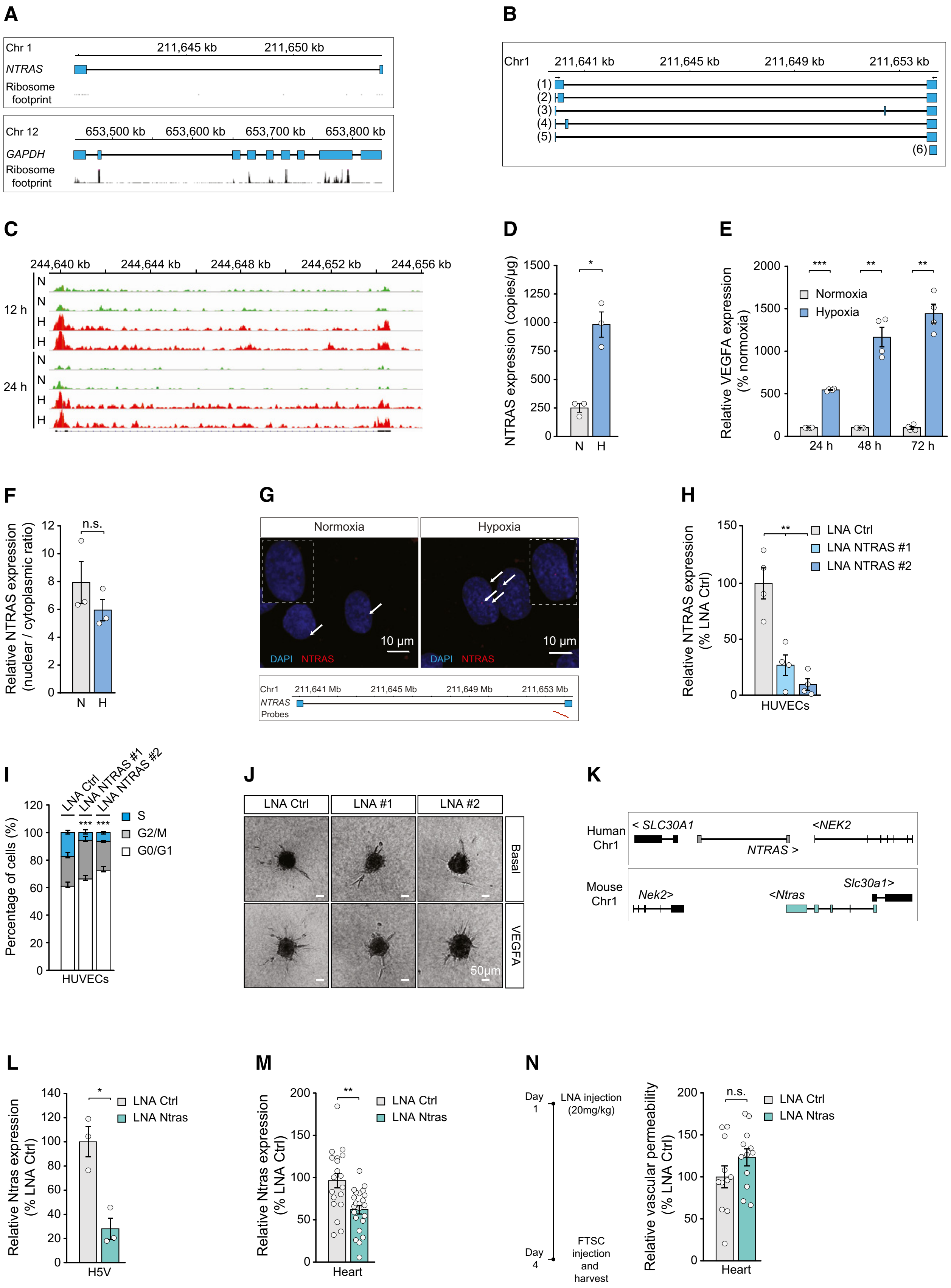
A
Chr 1
211,645 kb
211,650 kb
NTRAS
Ribosome footprint
Chr 12
653,500 kb
653,600 kb
653,700 kb
653,800 kb
GAPDH
Ribosome footprint
B
Chr1
211,641 kb
211,645 kb
211,649 kb
211,653 kb
(1)
(2)
(3)
(4)
(5)
(6)
C
244,640 kb
244,644 kb
244,648 kb
244,652 kb
244,656 kb
12 h
24 h
N
H
D
NTRAS expression (copies/µg)
N H
E
Relative VEGFA expression (% normoxia)
Normoxia
Hypoxia
24 h
48 h
72 h
F
Relative NTRAS expression (nuclear / cytoplasmic ratio)
n.s.
N H
G
Normoxia
Hypoxia
10 µm
DAPI NTRAS
Chr1
211,641 Mb
211,645 Mb
211,649 Mb
211,653 Mb
NTRAS
Probes
H
Relative NTRAS expression (% LNA Ctrl)
LNA Ctrl
LNA NTRAS #1
LNA NTRAS #2
HUVECs
I
LNA Ctrl
LNA NTRAS #1
LNA NTRAS #2
Percentage of cells (%)
S
G2/M
G0/G1
HUVECs
J
LNA Ctrl
LNA #1
LNA #2
Basal
VEGFA
50µm
K
Human Chr1
< SLC30A1
NTRAS >
<NEK2
Mouse Chr1
Nek2>
<Ntras
Slc30a1>
L
Relative Ntras expression (% LNA Ctrl)
LNA Ctrl
LNA Ntras
H5V
M
Relative Ntras expression (% LNA Ctrl)
LNA Ctrl
LNA Ntras
Heart
N
Day 1
LNA injection (20mg/kg)
Day 4
FTSC injection and harvest
Relative vascular permeability (% LNA Ctrl)
n.s.
LNA Ctrl
LNA Ntras
Heart

Figure EV1.

◀ **Figure EV2. NTRAS operates as splicing-regulatory lncRNA.**

A Relative expression of NTRAS in HUVECs and HeLa cells, determined by RT–qPCR (n = 4 independent biological replicates).
B RT–qPCR-based identification of accessible regions within NTRAS using RNase H-mediated cleavage of RNA–DNA heteroduplexes (DNA antisense oligonucleotides AS1 to AS5) in HUVEC cell lysate (n = 1). The oligonucleotide used for probe design is highlighted in red.
C Scheme illustrating the affinity selection of endogenous NTRAS–protein complexes for RNA and protein analysis.
D Sucrose density gradient ultracentrifugation showing the distribution of NTRAS and hnRNPL (protein). The dashed box indicates the fractions with the greatest overlap of both factors (n = 1).
E Illustration of the human NTRAS locus. Displayed is *NTRAS* (GRCh38.p13; RP11-354k1.1) and RBPmap-predicted hnRNPL binding motifs, described elsewhere (Smith *et al*, 2013).
F Western blot-based validation of NTRAS–hnRNPL interaction following antisense affinity selection of NTRAS in nuclear extracts from normoxic and hypoxic HUVECs (n = 4 independent biological replicates).
G Expression of NTRAS in control and NTRAS-silenced HUVECs used for RNA sequencing (n = 4 independent biological replicates).
H hnRNPL mRNA levels in control and hnRNPL-silenced HUVECs used for RNA sequencing (n = 3 independent biological replicates).
I RT–PCR-based analysis of CD55 intron 7 retention following hnRNPL/NTRAS double knockdown in HUVECs (n = 4 independent biological replicates). Representative agarose gel on the right.
J rMATs-based analysis of alternative splicing events upon silencing of lncRNA lncflow2 in HUVECs (n = 4 independent biological replicates) ES: Exon skipping, MXE: Mutually exclusive exons, A5SS: Alternative 5' splice site, A3SS: Alternative 3' splice site, RI: Retained intron.
K Validation of hnRNPU silencing in HUVECs by RT–qPCR (n = 4 independent biological replicates).
L RT–PCR-based analysis of TJP1 exon 20 inclusion in hnRNPU-silenced HUVECs (n = 4 independent biological replicates).

Data information: In (A, F–I, K, L), data are represented as mean ± SEM. n.s.: non-significant, $^{**}P < 0.01$, $^{***}P < 0.001$. (A, F–H, K, and L) two-tailed unpaired t-test, and (I) one-way ANOVA.
Source data are available online for this figure.

 © 2022 The Authors

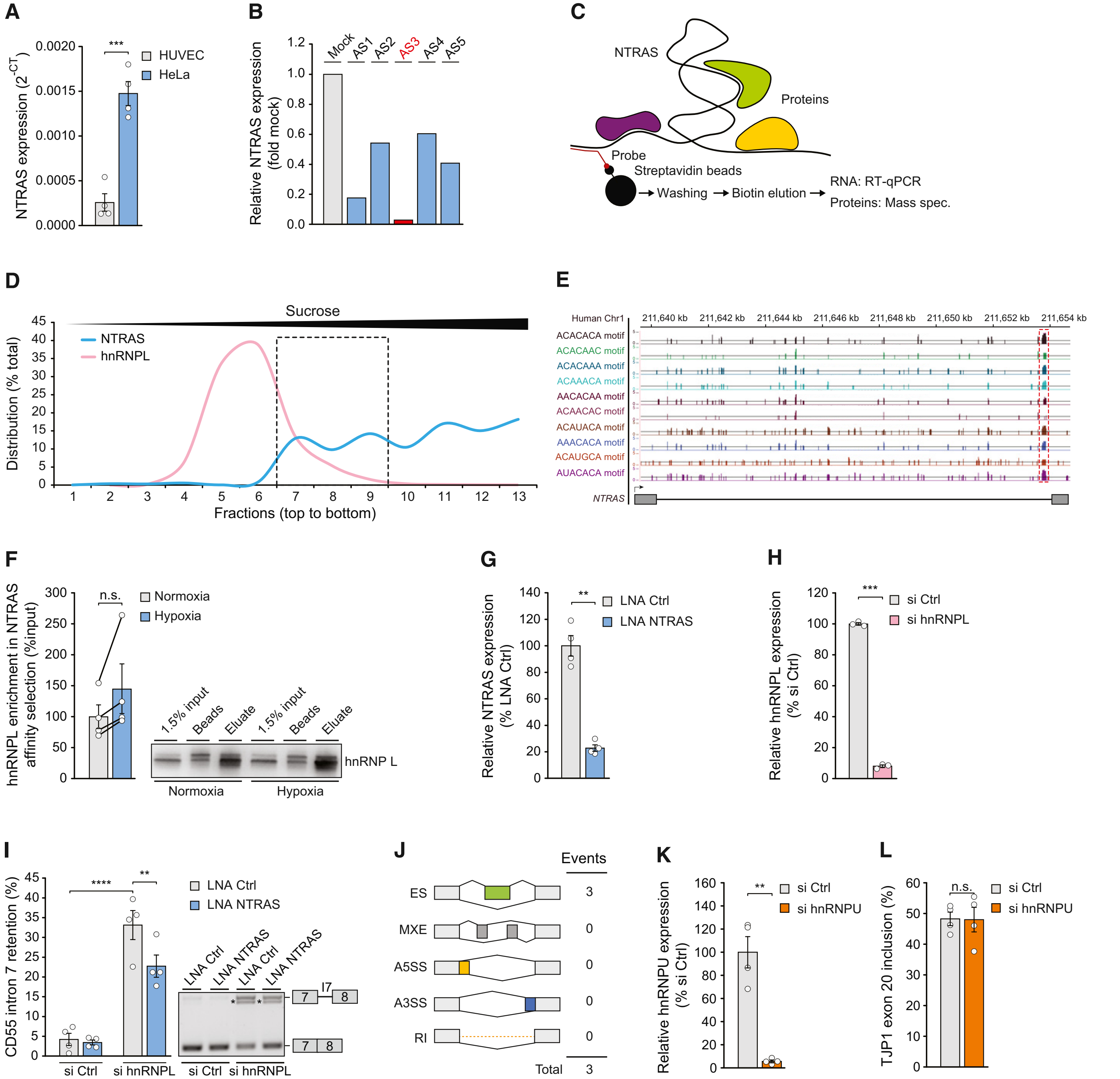
A
NTRAS expression (2^{-CT})
HUVEC
HeLa
***
B
Relative NTRAS expression (fold mock)
Mock
AS1
AS2
AS3
AS4
AS5
C
NTRAS
Proteins
Probe
Streptavidin beads
Washing
Biotin elution
RNA: RT-qPCR
Proteins: Mass spec.
D
Sucrose
NTRAS
hnRNPL
Distribution (% total)
Fractions (top to bottom)
E
Human Chr1
211,640 kb
211,642 kb
211,644 kb
211,646 kb
211,648 kb
211,650 kb
211,652 kb
211,654 kb
ACACACA motif
ACACAAC motif
ACACAAA motif
ACAAACA motif
AACACAA motif
ACAACAC motif
ACAUACA motif
AAACACA motif
ACAUGCA motif
AUACACA motif
NTRAS
F
hnRNPL enrichment in NTRAS affinity selection (%input)
n.s.
Normoxia
Hypoxia
1.5% input
Beads
Eluate
hnRNP L
G
Relative NTRAS expression (% LNA Ctrl)
**
LNA Ctrl
LNA NTRAS
H
Relative hnRNPL expression (% si Ctrl)
***
si Ctrl
si hnRNPL
I
CD55 intron 7 retention (%)
****
**
LNA Ctrl
LNA NTRAS
si Ctrl
si hnRNPL
I7
J
Events
ES 3
MXE 0
A5SS 0
A3SS 0
RI 0
Total 3
K
Relative hnRNPU expression (% si Ctrl)
**
si Ctrl
si hnRNPU
L
TJP1 exon 20 inclusion (%)
n.s.
si Ctrl
si hnRNPU

Figure EV2.

Figure EV3. NTRAS controls TJP1 splicing and endothelial barrier function.

A RT–qPCR-based analysis of TJP1 mRNA expression in hnRNPL-silenced HUVECs (*n* = 7 independent biological replicates).
B Relative expression of BCL2, CASP3, CASP6, and CASP9 mRNA in hnRNPL-silenced HUVECs (*n* = 2 independent biological replicates).
C RT–qPCR-based analysis of TJP1 mRNA expression in NTRAS-silenced HUVECs (*n* = 4 independent biological replicates).
D RT–PCR-based analysis of Tjp1 exon 20 inclusion in control and Ntras-silenced murine CMT93 epithelial cells (*n* = 3 independent biological replicates).
E RT–qPCR-based validation of RNase H-mediated NTRAS degradation in HeLa nuclear extracts used for *in vitro* splicing (*n* = 4 independent biological replicates).
F RT–qPCR-based validation of NTRAS overexpression (*n* = 4 independent biological replicates).
G Co-precipitation of NTRAS in anti-hnRNPL RIPs, using nuclear lysates from control and NTRAS-overexpressing cells (*n* = 4 independent biological replicates).
H RT–PCR-based validation of NTRAS-CA_{16} motif overexpression (*n* = 6 independent biological replicates). Representative agarose gel on the right.
I Quantification of nuclear TJP1, comparing control and NTRAS-silenced HUVECs (*n* = 4 independent biological replicates). Representative micrographs are shown. Scale bars are 10 μm.
J Analysis of hnRNPL knockdown by western blot 72 h post-transfection of control or hnRNPL-targeting siRNAs (*n* = 4 independent biological replicates). Representative western blots on the right.
K *In vitro* permeability assays using FITC-dextran, comparing control and hnRNPL-silenced HUVECs (*n* = 6 independent biological replicates).
L *In vitro* permeability assays using FITC-dextran, comparing control and hnRNPU-silenced HUVECs (*n* = 4 independent biological replicates).
M *In vitro* permeability assays using FITC-dextran, comparing control SSO- and E20 SSO-transfected HUVECs (*n* = 5 independent biological replicates).

Data information: In (A, C–M) data are represented as mean ± SEM. n.s.: non-significant, *$P < 0.05$, **$P < 0.01$. (A–I) two-tailed unpaired *t*-test.
Source data are available online for this figure.

 © 2022 The Authors

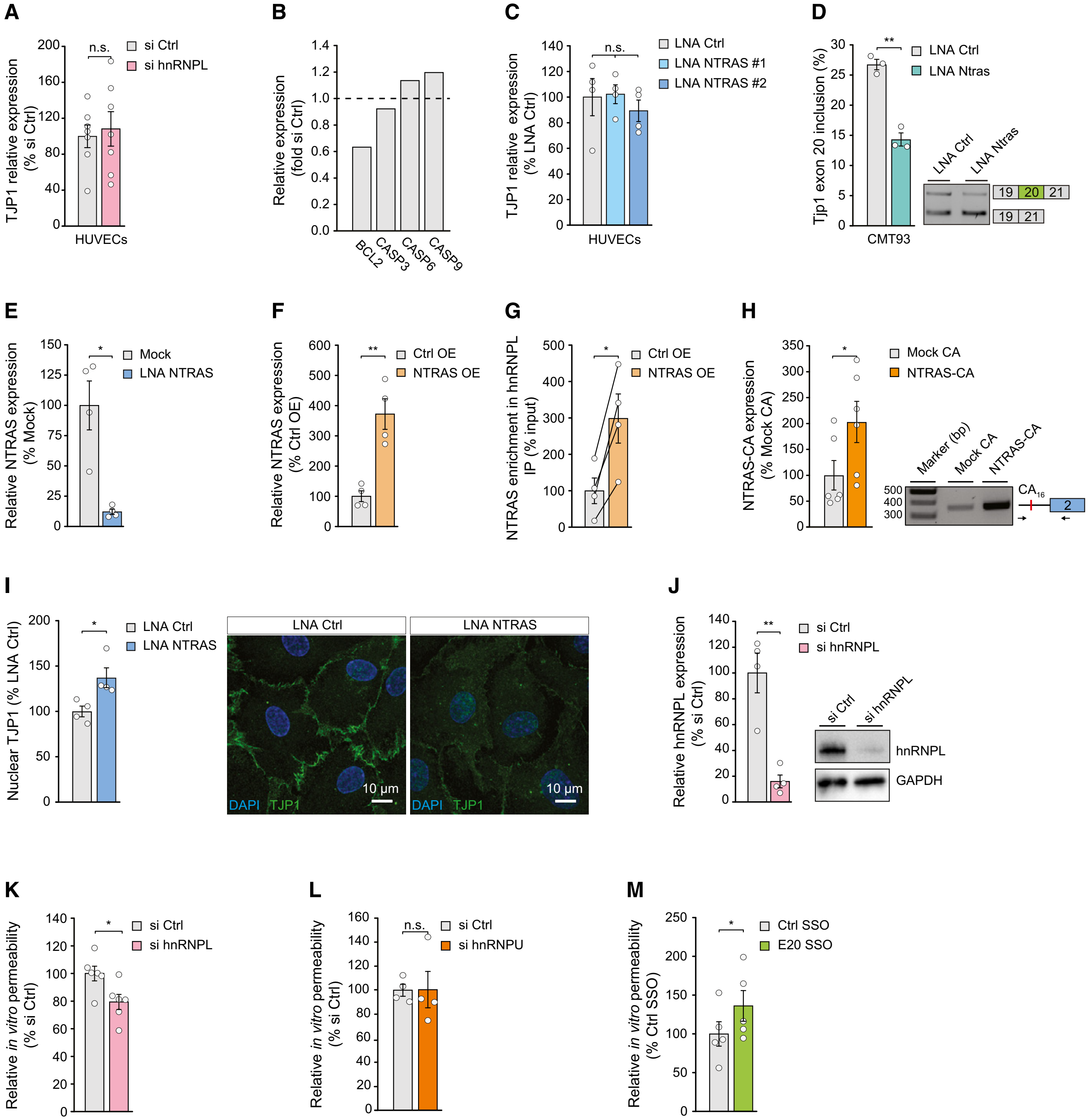
A
TJP1 relative expression (% si Ctrl)
n.s.
si Ctrl
si hnRNPL
HUVECs
B
Relative expression (fold si Ctrl)
BCL2
CASP3
CASP6
CASP9
C
TJP1 relative expression (% LNA Ctrl)
n.s.
LNA Ctrl
LNA NTRAS #1
LNA NTRAS #2
HUVECs
D
Tjp1 exon 20 inclusion (%)
LNA Ctrl
LNA Ntras
CMT93
19 20 21
19 21
E
Relative NTRAS expression (% Mock)
Mock
LNA NTRAS
F
Relative NTRAS expression (% Ctrl OE)
Ctrl OE
NTRAS OE
G
NTRAS enrichment in hnRNPL IP (% input)
Ctrl OE
NTRAS OE
H
NTRAS-CA expression (% Mock CA)
Mock CA
NTRAS-CA
Marker (bp)
CA16
I
Nuclear TJP1 (% LNA Ctrl)
LNA Ctrl
LNA NTRAS
DAPI TJP1
10 µm
J
Relative hnRNPL expression (% si Ctrl)
si Ctrl
si hnRNPL
hnRNPL
GAPDH
K
Relative in vitro permeability (% si Ctrl)
si Ctrl
si hnRNPL
L
Relative in vitro permeability (% si Ctrl)
n.s.
si Ctrl
si hnRNPU
M
Relative in vitro permeability (% Ctrl SSO)
Ctrl SSO
E20 SSO

Figure EV3.

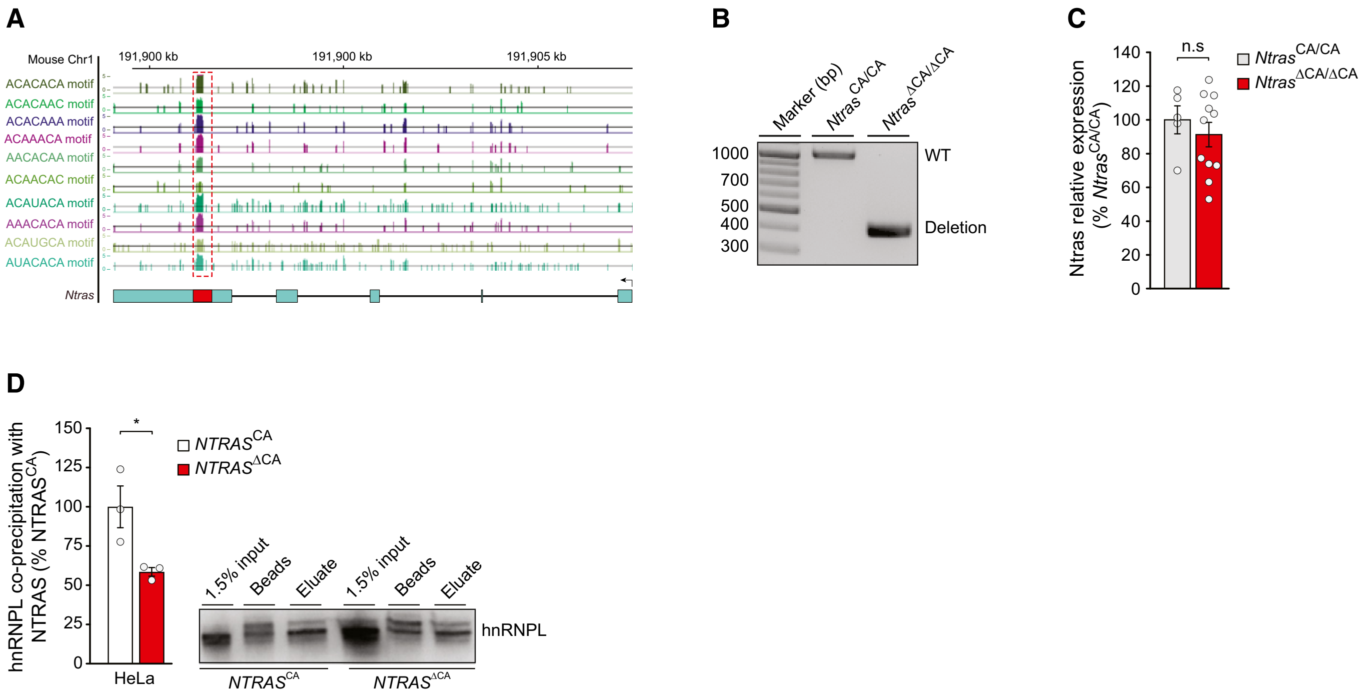

Figure EV4. Characterization of the Ntras CA-repeat *in vivo*.

A Illustration of the murine *Ntras* locus. Displayed is *Ntras* (GRCm38/mm10; 1700034H15) and RBPmap-predicted hnRNPL binding motifs, described elsewhere (Smith *et al*, 2013).

B Genotyping results confirming the genomic deletion of the hnRNPL binding motif in *Ntras*$^{\Delta CA/\Delta CA}$ mice.

C RT–qPCR-based analysis of Ntras expression in *Ntras*$^{CA/CA}$ and *Ntras*$^{\Delta CA/\Delta CA}$ mice (n = 5–11 mice per group).

D Western blot-based analysis of the NTRAS–hnRNPL interaction following antisense affinity selection of NTRAS in nuclear fractions from *NTRAS*^{CA} controls and *NTRAS*$^{\Delta CA}$ HeLa cells (n = 3 independent biological replicates). Representative western blot on the right.

Data information: In (C, D), data are represented as mean ± SEM. n.s.: non-significant, *$P < 0.05$. (C, D) two-tailed unpaired t-test.
Source data are available online for this figure.

© 2022 The Authors
