# Supplementary material for: The splicing‐regulatory lncRNA NTRAS sustains vascular integrity
Source: EMBO Rep. 2022 May 8;23(6):e54157. doi: 10.15252/embr.202154157 (PMC9171682; doi:10.15252/embr.202154157)
Supplement: Supplementary file 12 [file EMBR-23-e54157-s004.pdf]

EMBO reports

The splicing-regulatory lncRNA NTRAS sustains vascular integrity

Youssef Fouani, Luisa Kirchhof, Laura Stanicek, Guillermo Luxán, Andreas Heumüller, Andrea Knau, Ariane Fischer, Kavi Devraj, David John, Philipp Neumann, Albrecht Bindereif, Reinier Boon, Stefan Liebner, Ilka Wittig, Carolin Mogler, Madina Karimova, Stefanie Dimmeler, and Nicolas Jaé
DOI: 10.15252/embr.202154157

Corresponding author(s): Nicolas Jaé (jae@med.uni-frankfurt.de)

Review Timeline:

Submission Date:	14th Oct 21
Editorial Decision:	27th Nov 21
Revision Received:	23rd Dec 21
Editorial Decision:	10th Jan 22
Revision Received:	17th Feb 22
Accepted:	12th Apr 22

Editor: Bernd Pulverer

Transaction Report:

Please note that the manuscript was previously reviewed at another journal outside EMBO press and the reports were taken into account in the decision making process at EMBO Reports. Since the original reviews are not subject to EMBO Press' transparent review process policy, the reports and author response cannot be published.

(Note: With the exception of the correction of typographical or spelling errors that could be a source of ambiguity, letters and reports are not edited. Depending on transfer agreements, referee reports obtained elsewhere may or may not be included in this compilation. Referee reports are anonymous unless the Referee chooses to sign their reports.)

1st Editorial Decision 27th Nov 2021

Dear Dr. Jaé

Thank you for the submission of your revised manuscript to EMBO reports. As already discussed, we received the comments of our arbitrating subject expert who evaluated your response to the previous referee comments.

Thank you for your response - would like to invite a final revision which addresses the following issues:

1) Nomenclature: the use of both ZO-1 or TGP1a is confusing for the general reader. It is fine to stick to the latter, but please refer to both names in the abstract and explain the name decision at firt use in the manuscript.
2) Please explain in more detail in the manscript the refutation aspect to Lv et al. w.r.t transcriptional regulation vs. splicing. Include figs 1a-c 'for reviewers' (see also ref 2#13)
3) Please ensure the manuscript includes a clear explanation of the '2 models' to explain TJP1 splicing regulation holistically (sequestration & recruitment cf. ref 1 #3).
4) Include control Fig 3A-D 'for reviewers' that addresses ref. 1#4 as well as ref 2#9.
5) It is Ok to remove the HIF1 expression regulation data (ref 2#1), but it would appear reasonable to include the induction of hnRNP-NTRAS interaction data 'fig 4D for reviewers' (cf. ref 2 #7 & #12)
6) Ref 2 #2, #3, #5, #6, #8, #10, #11, #14, and #15-18, #20-21 are addressed
7) Ref 2 #4: include argument in manuscript with fig 5A 'for reviewers'.
8) Include the explanation to ref 2#19 in the manuscript.

With reference to the pre-decision discussion od the arbitrating referee's comments, please proceed as suggested to:
(i) assess TJP1 pre-mRNA splicing in vitro upon RNaseH-mediated degradation of NTRAS including the control.
(ii) tone down generalized mechanism of action for the splicing regulatory function of NTRAS-hnRNPL.

Please note that we would include the p-b-p response to the three referees and our advisor and your response to her/him in the transparent peer review process file.

Please include a completed 'authors checklist' upon resubmission.

We typically advise for revised manuscripts to be submitted within three months. Please contact us if a 3-months time frame is not sufficient for the revisions so that we can discuss this further.

Please include:
1) A data availability section providing access to data deposited in public databases is missing. If you have not deposited any data, please add a sentence to the data availability section that explains that.
2) Your manuscript contains underpowered or misapplied statistics. - the name of the statistical test used to generate error bars and P values,
- the number (n) of independent experiments (please specify technical or biological replicates) underlying each data point,
- the nature of the bars and error bars (s.d., s.e.m.),
- If the data are obtained from n {less than or equal to} 2, use scatter blots showing the individual data points.
Discussion of statistical methodology can be reported in the materials and methods section, but figure legends should contain a basic description of n, P and the test applied.

When submitting your revised manuscript, please carefully review the instructions that follow below. Failure to include requested items will delay the evaluation of your revision.

1) a .docx formatted version of the manuscript text (including legends for main figures, EV figures and tables). Please make sure that the changes are highlighted to be clearly visible.

2) individual production quality figure files as .eps, .tif, .jpg (one file per figure). See https://wol-prod-cdn.literatumonline.com/pb-assets/embo-site/EMBOPress_Figure_Guidelines_061115-1561436025777.pdf for more info on how to prepare your figures.

3) We replaced Supplementary Information with Expanded View (EV) Figures and Tables that are collapsible/expandable online. A maximum of 5 EV Figures can be typeset. EV Figures should be cited as 'Figure EV1, Figure EV2" etc... in the text and their respective legends should be included in the main text after the legends of regular figures.

- For the figures that you do NOT wish to display as Expanded View figures, they should be bundled together with their legends in a single PDF file called *Appendix*, which should start with a short Table of Content. Appendix figures should be referred to in the main text as: "Appendix Figure S1, Appendix Figure S2" etc. See detailed instructions regarding expanded view here: <https://www.embopress.org/page/journal/14693178/authorguide#expandedview>

- Additional Tables/Datasets should be labeled and referred to as Table EV1, Dataset EV1, etc. Legends have to be provided in

a separate tab in case of .xls files. Alternatively, the legend can be supplied as a separate text file (README) and zipped together with the Table/Dataset file.

4) a .docx formatted letter INCLUDING the reviewers' reports and your detailed point-by-point responses to their comments. As part of the EMBO Press transparent editorial process, the point-by-point response is part of the Review Process File (RPF), which will be published alongside your paper.

5) a complete author checklist, which you can download from our author guidelines <https://www.embopress.org/page/journal/14693178/authorguide>. Please insert information in the checklist that is also reflected in the manuscript. The completed author checklist will also be part of the RPF.

6) Please note that all corresponding authors are required to supply an ORCID ID for their name upon submission of a revised manuscript (<https://orcid.org/>). Please find instructions on how to link your ORCID ID to your account in our manuscript tracking system in our Author guidelines <https://www.embopress.org/page/journal/14693178/authorguide#authorshipguidelines>

7) Before submitting your revision, primary datasets produced in this study need to be deposited in an appropriate public database (see https://www.embopress.org/page/journal/14693178/authorguide#datadeposition). Please remember to provide a reviewer password if the datasets are not yet public. The accession numbers and database should be listed in a formal "Data Availability" section placed after Materials & Method (see also https://www.embopress.org/page/journal/14693178/authorguide#datadeposition). Please note that the Data Availability Section is restricted to new primary data that are part of this study. * Note - All links should resolve to a page where the data can be accessed. *
If your study has not produced novel datasets, please mention this fact in the Data Availability Section.

8) We would also encourage you to include the source data for figure panels that show essential data. Numerical data should be provided as individual .xls or .csv files (including a tab describing the data). For blots or microscopy, uncropped images should be submitted (using a zip archive if multiple images need to be supplied for one panel). Additional information on source data and instruction on how to label the files are available at <https://www.embopress.org/page/journal/14693178/authorguide#sourcedata>.

9) Our journal also encourages inclusion of *data citations in the reference list* to directly cite datasets that were re-used and obtained from public databases. Data citations in the article text are distinct from normal bibliographical citations and should directly link to the database records from which the data can be accessed. In the main text, data citations are formatted as follows: "Data ref: Smith et al, 2001" or "Data ref: NCBI Sequence Read Archive PRJNA342805, 2017". In the Reference list, data citations must be labeled with "[DATASET]". A data reference must provide the database name, accession number/identifiers and a resolvable link to the landing page from which the data can be accessed at the end of the reference. Further instructions are available at https://www.embopress.org/page/journal/14693178/authorguide#referencesformat

10) Regarding data quantification (see Figure Legends: https://www.embopress.org/page/journal/14693178/authorguide#figureformat)

11) Please also include scale bars in all microscopy images.

As part of the EMBO publication's Transparent Editorial Process, EMBO reports publishes online a Review Process File (RPF) to accompany accepted manuscripts. This File will be published in conjunction with your paper and will include the referee reports, your point-by-point response and all pertinent correspondence relating to the manuscript.

You are able to opt out of this by letting the editorial office know (emboreports@embo.org). If you do opt out, the Review Process File link will point to the following statement: "No Review Process File is available with this article, as the authors have chosen not to make the review process public in this case."

I look forward to seeing a revised form of your manuscript when it is ready. Please use this link to submit your revision: https://embor.msubmit.net/cgi-bin/main.plex

Yours sincerely,

Bernd Pulverer

~~~~~~~~~~~~~~~~~~~~~~~~~~~~~~~~
Bernd Pulverer, Ph.D.
~~~~~~~~~~~~~~~~~~~~~~~~~~~~~~~~

Chief Editor, EMBO Reports
EMBO
Meyerhofstrasse 1, D-69117 Heidelberg
Tel: +4962218891501
bernd.pulverer@embo.org
~~~~~~~~~~~~~~~~~~~~~~~~~~~~~~~~~~

Referee #1:

I have carefully read the manuscript and point-by-point rebuttal and I think authors have really made a big effort to address all reviewers concerns. However, there is still a small issue, the biggest concern is whether it was demonstrated that NTRAS effect on splicing is via sequestration (or not) of hnRNPL. The data around TJP1 is convincing, but no other examples are given and the impact in splicing upon OE of the lncRNA (or just the CA motif) is not even close to the one obtained when hnRNPL is depleted, suggesting that this might not be the major mechanism of splicing regulation. Actually, Reviewer 1 asked for some interesting rescue experiments that were not actually done. Other experiments were proposed by the authors, but no rescue was done to prove the mechanistic relationship between NTRAS and hnRNPL. I think this is important.

Finally, when looking for more examples, we have to go to suppl fig S2 and the effects are not very convincing either. So I am not sure such mechanism can be generalised, the genome-wide data is not properly validated. If authors provide more examples of hnRNPL sponging, the manuscript can be published. Else it needs to be tone down.
~~~~~~~~~~~~~~~~~~~~~~~~~~~~~~~~~~

Point-by-point response EMBOR-2021-54157-T

1) Nomenclature: the use of both ZO-1 or TGP1a is confusing for the general reader. It is fine to stick to the latter, but please refer to both names in the abstract and explain the name decision at firt use in the manuscript.

We followed the editor's advice and consistently used the HUGO Gene Nomenclature Committee approved name TJP1 throughout the revised manuscript. ZO-1, as alternative name, is mentioned in the abstract. The relevant section now reads: "Biochemical analysis revealed that NTRAS, through its CA-dinucleotide repeat motif, sequesters the splicing regulator hnRNPL to control alternative splicing of tight junction protein 1 (TJP1; also named zona occludens 1, ZO-1) pre-mRNA."

In addition, the intuitive name decision for TJP1 is made at first use in the introduction. The relevant section now reads: "Tight junctions are multiprotein junctional complexes comprising the three major transmembrane proteins occludin, claudins, and junction adhesion molecules, which associate with different peripheral membrane proteins such as tight junction protein 1 (TJP1, also named ZO-1). Encoded by the TJP1 gene, this multidomain protein is located on the intracellular side of the plasma membrane to anchor the transmembrane junctional proteins to the actin component of the cytoskeleton (Campbell, Maiers and DeMali, 2017)."

2) Please explain in more detail in the manscript the refutation aspect to Lv et al. w.r.t transcriptional regulation vs. splicing. Include figs 1a-c 'for reviewers' (see also ref 2#13)

Following the editor's suggestion, we addressed the findings by Lv et al. and in this context integrated all above mentioned figures. The revised paragraph now reads: "In this context, a recently reported regulation of TJP1 total expression levels (and apoptosis-related proteins) by hnRNPL in epithelial cells (Lv et al., 2017) could not be observed for endothelial cells (Figure EV3A, B). Likewise, NTRAS silencing in endothelial cells did not influence TJP1 total mRNA levels (Figure EV3C). However, exon 20 splicing regulation by NTRAS was also evident in the epithelium (Figure EV3D)."

3) Please ensure the manuscript includes a clear explanation of the '2 models' to explain TJP1 splicing regulation holistically (sequestration & recruitment cf. ref 1 #3).

Based on the limitation of our data to explain the synergistic splicing events (recruitment model), the recommendation to re-focus the revised manuscript on the mechanism of TJP1 splicing, and with reference to the pre-decision discussion with the editor, we removed data and figures emphasizing a putative recruitment model. This applies to original figures 2I and 2J. The confirmation of additional, NTRAS-hnRNPL co-regulated pre-mRNAs was moved to Appendix figure 1B-F and is now cited in the revised discussion which deals with the interesting aspects that 1. NTRAS-hnRNPL might regulate additional transcripts beyond TJP1 and 2. This might be achieved by other mechanisms than hnRNPL sequestration. The revised paragraph comprises lines 275 to 291.

4) Include control Fig 3A-D 'for reviewers' that addresses ref. 1#4 as well as ref 2#9.

To underline the specificity of our identified NTRAS-hnRNPL axis, we followed the editor's advice and included an additional lncRNA control for the assessment of genome-wide splicing regulation. This

new data is shown in Figure EV2J of the revised manuscript. In this context, we also demonstrated the specificity of hnRNPL on TJP1 exon 20 inclusion by silencing of the splicing factor hnRNPU which failed to reproduce the outcome of hnRNPL silencing. This supporting data is now shown as new Figures EV2K, L. The combined section now reads: "Of note, silencing of an unrelated control lncRNA and hnRNPU, a heterogeneous nuclear ribonucleoprotein not associated with NTRAS, failed to regulate TJP1 exon 20 inclusion rates (Figure EV2J-L)."

Finally, we included data demonstrating unchanged endothelial permeability upon silencing of hnRNPU, see Figure EV3L of the revised manuscript. The paragraph addressing this new data reads: "In contrast, silencing of hnRNPL (Figure EV3J) specifically augmented barrier function (Fig 3H and Figure EV3K), whereas silencing of the non-specific splicing factor hnRNPU had no effect (Figure EV3L)."

5) It is Ok to remove the HIF1 expression regulation data (ref 2#1), but it would appear reasonable to include the induction of hnRNP-NTRAS interaction data 'fig 4D for reviewers' (cf. ref 2 #7 & #12)

As suggested by the editor, we removed the HIF1 data from the revised manuscript and included data on the augmented interaction between NTRAS and hnRNPL following hypoxia. This new data is shown in Figure EV2F of the revised manuscript.

6) Ref 2 #2, #3, #5, #6, #8, #10, #11, #14, and #15-18, #20-21 are addressed

Nothing to add.

7) Ref 2 #4: include argument in manuscript with fig 5A 'for reviewers'.

As requested by the editor, we included the sucrose density gradient ultracentrifugation showing the overlapping distribution of NTRAS and hnRNPL as new Figure EV2D of the revised manuscript. The rearranged paragraph reads: "Given that hnRNPL is a highly expressed protein (Beck et al., 2011) whereas NTRAS is rather a low abundant lncRNA, we questioned the stoichiometry of both factors. To this end, we deployed density gradient ultracentrifugation (Figure EV2D) revealing that the majority of hnRNPL (~ 79 %) is not bound to NTRAS. However, a major fraction of NTRAS co-sediments with hnRNPL, supporting the supposed interaction of both factors. This result is in line with the circumstance that hnRNPL is engaged in a multitude of different RNA-binding processes, whereas the association with NTRAS might be involved in fine tuning a specific subset of hnRNPL-mediated processes. In addition, in silico analysis of the NTRAS sequence revealed several CA-rich hnRNPL binding motifs and strikingly a prominent bona fide hnRNPL binding site in the form of a CA16 repeat sequence proximal to the 3' splice site of the predominantly retained intron 2 (Figure EV2E). Therefore, it might be reasonably assumed that the presence of multiple hnRNPL binding motifs within NTRAS will compensate for the unfavorable stoichiometry between both factors. Finally, RNA immunoprecipitation (Fig 2D) and RNA affinity selection followed by western blotting (Figure EV2F) unequivocally validated the interaction between NTRAS and hnRNPL. Furthermore, such interaction was enhanced under hypoxia-mediated NTRAS upregulation, corroborating the aforementioned data (Figure EV2F). In summary, our results suggest that NTRAS exists as a constituent of an hnRNPL-containing ribonucleoprotein complex in the nucleus.

8) Include the explanation to ref 2#19 in the manuscript.

We followed the editor's advice and clarified the usage of the two-exon mini-gene construct for our in vitro splicing assays. The revised passage now reads: "First, we assessed the in vitro splicing efficiency of a TJP1 minigene construct upon NTRAS depletion in splicing competent nuclear extract. Since the in vitro transcription of an exon 19-20-21 TJP1 minigene proved to be inefficient, we deployed a previously described construct, comprising the constitutive exon 19, intron 19 (which contains the hnRNPL binding motifs), and the alternative exon 20 (Fig. 3A) (Heiner et al., 2010). RNase H-mediated NTRAS degradation in nuclear extracts prior to splicing (Figure EV3E) significantly diminished the splicing efficiency of the TJP1 exon 19-20 minigene (Fig 3B). Strikingly, this effect could be rescued by the addition of an in vitro transcribed NTRAS fragment, harboring the CA_{16} dinucleotide repeat, prior to splicing (Fig 3B)."

With reference to the pre-decision discussion od the arbitrating referee's comments, please proceed as suggested to:

(i) assess TJP1 pre-mRNA splicing in vitro upon RNaseH-mediated degradation of NTRAS including the control.

We successfully rescued the impaired splicing efficiency of our TJP1 mini-gene, induced by RNaseH-mediated degradation of NTRAS, by add-back of an NTRAS fragment harboring the major hnRNPL binding motif. This new data demonstrates the binding competition between hnRNPL, NTRAS and the TJP1 pre-mRNA and is shown in the revised manuscript as new Figure 3B, thereby replacing the previously shown in vitro splicing data. The corresponding section reads: "RNase H-mediated NTRAS degradation in nuclear extracts prior to splicing (Figure EV3E) significantly diminished the splicing efficiency of the TJP1 exon 19-20 minigene (Fig 3B). Strikingly, this effect could be rescued by the addition of an in vitro transcribed NTRAS fragment, harboring the CA_{16} dinucleotide repeat, prior to splicing (Fig 3B)."

(ii) tone down generalized mechanism of action for the splicing regulatory function of NTRAS-hnRNPL.

In accordance with our response to comment 3), we re-focused our revised manuscript on the molecular mechanism of NTRAS-hnRNPL regulating TJP1 exon 20 usage and eventually endothelial permeability.

To this end, we removed most of the data addressing a general splicing regulatory mechanism, specifically the original figures 2I and 2J. However, to indicate that our observed splicing regulatory processes are not strictly limited to TJP1 exon 20, we moved the additionally validated NTRAS-hnRNPL splice substrates to Appendix figure 1B-F and chose to address the notion that both factors might be part of a more complex splicing network in the discussion; see lines 275 to 291.

Please include:

1) A data availability section providing access to data deposited in public databases is missing. If you have not deposited any data, please add a sentence to the data availability section that explains that.

A data availability section is provided in the revised manuscript and RNA sequencing and mass spectrometry data were deposited in a publicly available repository. RNA sequencing data can be accessed via the identifier E-MTAB-11311, and the mass spectrometry data via PXD030620.

2) Your manuscript contains underpowered or misapplied statistics. - the name of the statistical test used to generate error bars and P values, - the number (n) of independent experiments (please specify technical or biological replicates) underlying each data point, - the nature of the bars and error bars (s.d., s.e.m.), - If the data are obtained from n {less than or equal to} 2, use scatter blots showing the individual data points. Discussion of statistical methodology can be reported in the materials and methods section, but figure legends should contain a basic description of n, P and the test applied.

We revised our statistics section, explaining the nature of bar graphs, error bars and statistical tests for generating p values. In addition, these information are given in the figure legends, together with an experiment-specific statement regarding the number of biological replicates. Finally, we included individual data points for every experiment, or when not possible (e.g. genome-wide sequencing data), relevant data points (here: TJP1 exon 20 usage in NTRAS- and hnRNPL-silenced HUVECs) were extracted and are shown separately in Appendix figure 1A.

Dear Dr. Jaé

Thank you for the re-submission of your manuscript to EMBO reports. We have now received the report of our arbitrating referee (below)

As you will see, the referee acknowledges clear progress, but s/he continues to raise significant issues on key elements of the data. Indeed, they do not view the current evidence for the functional impact of the sequestration compelling. Publication is contingent on presenting compelling effects and a well supported mechanism.

In particular, the referee noted the impact of NTRAS depletion is not convincing and suggested the rescue with the endogenous gene, not a minigene.

The referee is not convinced by the statistical significance of the new data: 'Appendix figure panel C cannot be a two star significance. Or Appendix F. Or Fig. 3B as mentioned before, or 3D, 4G, etc...if data points overlap, how come it is significant?'

The referee also raises a potential discrepancies:
1) re. Appendix Figure 1: 'KD of NTRAS represents an overexpression of hnRNPL. How come the KD of the lncRNA impacts splicing in the same direction as KD of the splicing factor? How come TJP1 ex20 changes are not significant.
2) in Fig EV2.J, it seems like only 3 skipped exons are impacted upon silencing of the lncRNA, but then in appendix 6 exons are analysed.

The refere concludes: 'The data is not strong enough and the whole model just stands for one exon, which I do not understand why if it is a sequestration mechanism...where the specificity comes from?

[full report below]

We typically only undertake a single round of substantive experimental revision. This is to avoid excessive delays to publication and frustration. In this case, we feel that the revision have moved the manuscript in a significant manner, but we cannot publish the data unless the referee is substantially more convinced on key points. We are exceptionally willing to undertake a further round of revision if the key points are addressabel, but fear that this will not be possible in a sufficiently short time frame - if at all. As such, we would at this point understand completely of publication elsewhere is sought. We will be happy to forward the referee reports in hand to another journal if you decide that is useful.
If you think a further revision may be realistic, I suggest to send a revision plan first to avoid futile delays that may compromize your research project.

In case a revision is pursued, there are a number of minor publishing technical issues that remain open:
1) We strongly encourage addition of source data, minimally for all key data
2) Please add Arrayexpress accession codes
3) REFERENCE FORMAT: Many are only 1 author et al.
4): Please ensure Grant numbers and orgs a fully listed - changed after publication are only exceptionally possible
5) Appendix Fix S1 callout needs correcting, the 'S' is missing.
6) DATASET EV LEGENDS: The tables should be named Dataset EV# and the legends need adding. The legends need removing from the manuscript file.
7) APPENDIX 1 FILE WITH ToC: A ToC is missing. The legends need removing from the manuscript file.
8) please add a SYNOPSIS IMAGE, if available
9) Below the Abstract is a Significance statement - this is not jnls format
10) Headings for the Expanded View Figure Legends are missing.

Yours sincerely,

Bernd Pulverer

~~~~~~~~~~~~~~~~~~~~~~~~~~~~~~~~
Bernd Pulverer, Ph.D.
Chief Editor, EMBO Reports
EMBO
Meyerhofstrasse 1, D-69117 Heidelberg
Tel: +4962218891501
bernd.pulverer@embo.org
~~~~~~~~~~~~~~~~~~~~~~~~~~~~~~~~

PS:

General guidelines FYI:
When submitting your revised manuscript, please carefully review the instructions that follow below. Failure to include requested items will delay the evaluation of your revision.

1) a .docx formatted version of the manuscript text (including legends for main figures, EV figures and tables). Please make sure that the changes are highlighted to be clearly visible.

2) individual production quality figure files as .eps, .tif, .jpg (one file per figure). See https://wol-prod-cdn.literatumonline.com/pb-assets/embo-site/EMBOPress_Figure_Guidelines_061115-1561436025777.pdf for more info on how to prepare your figures.

3) We replaced Supplementary Information with Expanded View (EV) Figures and Tables that are collapsible/expandable online. A maximum of 5 EV Figures can be typeset. EV Figures should be cited as 'Figure EV1, Figure EV2" etc... in the text and their respective legends should be included in the main text after the legends of regular figures.

- For the figures that you do NOT wish to display as Expanded View figures, they should be bundled together with their legends in a single PDF file called *Appendix*, which should start with a short Table of Content. Appendix figures should be referred to in the main text as: "Appendix Figure S1, Appendix Figure S2" etc. See detailed instructions regarding expanded view here: <https://www.embopress.org/page/journal/14693178/authorguide#expandedview>

- Additional Tables/Datasets should be labeled and referred to as Table EV1, Dataset EV1, etc. Legends have to be provided in a separate tab in case of .xls files. Alternatively, the legend can be supplied as a separate text file (README) and zipped together with the Table/Dataset file.

4) a .docx formatted letter INCLUDING the reviewers' reports and your detailed point-by-point responses to their comments. As part of the EMBO Press transparent editorial process, the point-by-point response is part of the Review Process File (RPF), which will be published alongside your paper.

5) a complete author checklist, which you can download from our author guidelines <https://www.embopress.org/page/journal/14693178/authorguide>. Please insert information in the checklist that is also reflected in the manuscript. The completed author checklist will also be part of the RPF.

6) Please note that all corresponding authors are required to supply an ORCID ID for their name upon submission of a revised manuscript (<https://orcid.org/>). Please find instructions on how to link your ORCID ID to your account in our manuscript tracking system in our Author guidelines <https://www.embopress.org/page/journal/14693178/authorguide#authorshipguidelines>

7) Before submitting your revision, primary datasets produced in this study need to be deposited in an appropriate public database (see https://www.embopress.org/page/journal/14693178/authorguide#datadeposition). Please remember to provide a reviewer password if the datasets are not yet public. The accession numbers and database should be listed in a formal "Data Availability" section placed after Materials & Method (see also https://www.embopress.org/page/journal/14693178/authorguide#datadeposition). Please note that the Data Availability Section is restricted to new primary data that are part of this study. * Note - All links should resolve to a page where the data can be accessed. *
If your study has not produced novel datasets, please mention this fact in the Data Availability Section.

8) We would also encourage you to include the source data for figure panels that show essential data. Numerical data should be provided as individual .xls or .csv files (including a tab describing the data). For blots or microscopy, uncropped images should be submitted (using a zip archive if multiple images need to be supplied for one panel). Additional information on source data and instruction on how to label the files are available at <https://www.embopress.org/page/journal/14693178/authorguide#sourcedata>.

9) Our journal also encourages inclusion of *data citations in the reference list* to directly cite datasets that were re-used and obtained from public databases. Data citations in the article text are distinct from normal bibliographical citations and should directly link to the database records from which the data can be accessed. In the main text, data citations are formatted as follows: "Data ref: Smith et al, 2001" or "Data ref: NCBI Sequence Read Archive PRJNA342805, 2017". In the Reference list, data citations must be labeled with "[DATASET]". A data reference must provide the database name, accession number/identifiers and a resolvable link to the landing page from which the data can be accessed at the end of the reference. Further instructions are available at https://www.embopress.org/page/journal/14693178/authorguide#referencesformat

10) Regarding data quantification (see Figure Legends: https://www.embopress.org/page/journal/14693178/authorguide#figureformat)

The following points must be specified in each figure legend:

- the name of the statistical test used to generate error bars and P values,

- the number (n) of independent experiments (please specify technical or biological replicates) underlying each data point,

- the nature of the bars and error bars (s.d., s.e.m.),

- If the data are obtained from n {less than or equal to} 2, use scatter blots showing the individual data points.

Discussion of statistical methodology can be reported in the materials and methods section, but figure legends should contain a basic description of n, P and the test applied.

- Please also include scale bars in all microscopy images.

We would also welcome the submission of cover suggestions, or motifs to be used by our Graphics Illustrator in designing a cover.

As part of the EMBO publication's Transparent Editorial Process, EMBO reports publishes online a Review Process File (RPF) to accompany accepted manuscripts. This File will be published in conjunction with your paper and will include the referee reports, your point-by-point response and all pertinent correspondence relating to the manuscript.

You are able to opt out of this by letting the editorial office know (emboreports@embo.org). If you do opt out, the Review Process File link will point to the following statement: "No Review Process File is available with this article, as the authors have chosen not to make the review process public in this case."

....

Referee #1:

The authors have significantly improved the manuscripts.

However I still have some concerns regarding the new data:

1) Fig. 3: The rescue is convincing, although I was expecting this to be done in the endogenous gene, not a minigene. Why aren't the authors providing the data from the endogenous gene? However the impact of NTRAS depletion is not convincing at all with all these overlapping points. I recall the effect to be clearer...

2) Appendix Figure 1: KD of NTRAS represents an overexpression of hnRNPL (since the lncRNA sequesters the splicing factor). How come the KD of the lncRNA impacts splicing in the same direction as KD of the splicing factor? Even more worrying, how come TJP1 ex20 changes are not significant (this concern is raised in the point before).

3) in Fig EV2.J, it seems like only 3 skipped exons are impacted upon silencing of the lncRNA, but then in appendix 6 exons are analysed. I do not understand the discrepancy.

4) Finally I am concerned about statistics. Appendix figure panel C cannot be a two star significance. Or Appendix F. Or Fig. 3B as mentioned before, or 3D, 4G, etc...if data points overlap, how come it is significant?

Overall, I am not sure the model is right. I believe NTRAS sequesters hnRNPL. That this has a biological impact, I am not that sure. The data is not strong enough and the whole model just stands for one exon, which I do not understand why if it is a sequestration mechanism...where the specificity comes from?

I think the authors need to clarify these points and show more consistent data.

Point-by-point response EMBOR-2021-54157V2

1. In particular, the referee noted the impact of NTRAS depletion is not convincing and suggested the rescue with the endogenous gene, not a minigene.

We thank the reviewer for this suggestion, however, want to clarify that based on the transcript length of TJP1, an *in vitro* splicing assay using the full-length TJP1 pre-mRNA is technically not feasible. Minigenes, in turn, proved to be valuable tools to specifically assess splicing patterns of interest. Of note, the TJP1 minigene deployed by us has been successfully used by others (e.g. Heiner et al., 2010) to identify and analyse the splicing repressive function of hnRNPL on TJP1 exon 20.

Finally, we would like to point out that we have already demonstrated the effect of the native endogenous RNA on splicing by overexpression of NTRAS in **Fig. 3F** of the manuscript.

2. The referee is not convinced by the statistical significance of the new data: 'Appendix figure panel C cannot be a two-star significance. Or Appendix F. Or Fig. 3B as mentioned before, or 3D, 4G, etc... if data points overlap, how come it is significant?'

Please note that we performed the appropriate statistical analysis for all figures with $n \geq 3$ biological replicates. The results confirm p-values below 0.05 **(see screen shots provided in Figure 1 for the reviewer)**, which is accepted as gold standard for concluding statistically significant differences. In addition, we are happy to provide the source data along with our submission, allowing the reviewer to confirm our analysis. Of note, columns with overlapping data points can be significantly different, if the statistical test used compares the means of the individual sample groups, which is e.g. the case for Student's t-tests. Finally, we want to state that the results shown in **Appendix Figure S1A-F** were primarily included to support / verify the $n = 2$ RNA sequencing results of **Fig. 2H**, where no statistical analysis is possible.

3. The referee also raises a potential discrepancies:

re. Appendix Figure 1: 'KD of NTRAS represents an overexpression of hnRNPL. How come the KD of the lncRNA impacts splicing in the same direction as KD of the splicing factor? How come TJP1 ex20 changes are not significant.

Response to the first part of the reviewer's comment: *"KD of NTRAS represents an overexpression of hnRNPL."*

We want to emphasize that we never showed, assumed, or suggested that NTRAS regulates hnRNPL expression positively or negatively. What the reviewer might have misunderstood in **Appendix Figure S1A** is that NTRAS silencing (blue column; LNA NTRAS) represses TJP1 exon 20 inclusion (please see axis-title) compared to the control condition (light grey; LNA Ctrl). On the other hand, hnRNPL silencing (pink column; si hnRNPL) enhances exon 20 inclusion compared to the control condition (dark grey; si Ctrl).

Response to the second part of the reviewers comment: *"How come the KD of the lncRNA impacts splicing in the same direction as KD of the splicing factor?"*

Indeed, we report some examples in which NTRAS knock down impacts splicing of pre-mRNAs in the same direction as knockdown of hnRNPL. However, we do not claim that these events are necessarily causally linked and the main intention of showing the data provided in the Appendix was to validate the RNA sequencing data. Of note, based on our recent correspondence with EMBO Reports and the agreement to focus on TJP1 splicing while toning down statements addressing transcriptome-wide splicing-regulatory mechanisms of NTRAS-hnRNPL, we decided to remove the complex transcriptome data from the revised manuscript. We believe that this greatly enhances the accessibility of our manuscript and while we agree that a detailed transcriptome-wide mechanistic analysis is thrilling, we consider this to be beyond the scope of our actual manuscript. Nevertheless, we hope that our sequencing data might be the starting point for follow-up studies, specifically dedicated to this very interesting mechanistic detail.

Response to the third statement: "How come TJP1 ex20 changes are not significant."

We have sequenced two biological replicates per condition. This study design precludes a statistical analysis but was meant to be hypothesis generating. Please note that the data on TJP1 exon 20 usage were subsequently validated by various experiments. E.g. **Fig. 2I** ($n = 7$, $p = 2.88E-05$); **Fig. 2J** ($n = 4$, $p < 0.000517$); **Fig. 3B** ($n = 7-12$, $p < 0.05$); **Fig. 3F** ($n = 8$, $p < 0.01$); **Fig. 4B** ($n = 8$, $p = 0.0035$); **Fig. 4E** ($n = 9-12$, $p = 0.04$); **Fig. EV3D** ($n = 3$, $p = 0.001037$). Together, the impact of NTRAS was confirmed by 6 independent experiments with multiple biological replicates both in human and mouse samples.

4. in Fig EV2.J, it seems like only 3 skipped exons are impacted upon silencing of the lncRNA, but then in appendix 6 exons are analysed.

We apologize for any misunderstandings and want to clarify that **Fig. EV2J** shows the overall differential splicing changes upon silencing of an unrelated lncRNA (lincflow2). In this analysis, only 3 skipped exons were affected compared to 131 exon skipping events upon NTRAS silencing (**Fig. 2G**).

The 6 exons analysed in the appendix, as the figure legend indicates, are examples of these NTRAS-regulated alternative splicing events.

5. Overall, I am not sure the model is right. I believe NTRAS sequesters hnRNPL. That this has a biological impact, I am not that sure. The data is not strong enough and the whole model just stands for one exon, which I do not understand why if it is a sequestration mechanism...where the specificity comes from?

Regarding the reviewer's concern, we specifically and exclusively propose a sequestration model for splicing of TJP1 exon 20. This model is based on ample mechanistic evidence and we eventually demonstrate the biological impact of NTRAS-hnRNPL-mediated regulation of TJP1 splicing.

A short summary of the key data supporting our conclusions is listed below and shown in **Figures 2-5 for the reviewer**:

For the sequestration of hnRNPL by NTRAS, please see **Figures 2 and 3 for the reviewer**:

Fig. 2A and B for the reviewer show the presence of bona fide hnRNPL binding motifs in human and mouse NTRAS transcripts along with numerous lower-ranking binding sites.

Fig. 2C and D for the reviewer prove reduced binding of hnRNPL to human and mouse NTRAS transcripts upon genomic deletion of the respective hnRNPL binding sites.

Fig. 3A-C for the reviewer show that **(A)** silencing of NTRAS enhances the direct association of TJP1 pre-mRNA and hnRNPL, while **(B)** overexpression of full length NTRAS or **(C)** the hnRNPL binding site of NTRAS diminishes this association. These results demonstrate that NTRAS competes with TJP1 pre-mRNA for hnRNPL binding.

For the specificity, please **see Figure 4 for the reviewer**:

Fig. 4A-F for the reviewer show that **(A)** silencing of hnRNPL but not of the **(B)** unrelated splicing factor hnRNPU affects TJP1 exon 20 splicing. Likewise, silencing of **(C)** NTRAS but not of the **(D)** unrelated lncRNA lincflow2 affects TJP1 exon 20 splicing. Moreover, **(E)** overexpression of the specific hnRNPL binding site of NTRAS or full-length NTRAS enhances TJP1 exon 20 inclusion, whereas **(F)** deletion of the hnRNPL binding site in vivo reduces exon 20 inclusion. Together these results demonstrate the specificity of hnRNPL and NTRAS in regulating TJP1 exon 20 usage.

For the biological impact, please **see Figure 5 for the reviewer**:

Fig. 5A-C for the reviewer show that the exclusive deletion of the hnRNPL binding site of NTRAS alone significantly **(A)** impairs retina vascularization, **(B)** cardiac vascular integrity and **(C)** enhances immune cells infiltration. In summary, we believe that this results clearly demonstrate a biological relevance of NTRAS.

Dr. Nicolas Jaé
Institute for Cardiovascular Regeneration
Germany

Dear Dr. Jaé,

Thank you for your detailed and informative response to the last round of per review. We have discussed your arguments and the latest changes introduced in revision. I am very pleased to accept your manuscript for publication in the next available issue of EMBO reports.

At the end of this email I include important information about how to proceed. Please ensure that you take the time to read the information and complete and return the necessary forms to allow us to publish your manuscript as quickly as possible.

We are using the CRediT author contribution format now, which allows for systematic, machine readable author contributions and thus more effective research assessment, while also allowing addition of any relevant further information for each author. We'll thus need to have your update authors contribution section updated.

As part of the EMBO publication's Transparent Editorial Process, EMBO reports publishes online a Review Process File to accompany accepted manuscripts. As you are aware, this File will be published in conjunction with your paper and will include the referee reports, your point-by-point response and all pertinent correspondence relating to the manuscript, including the last round of arbitrating advice and your rebuttal. For completeness and to allow the interested reader to follow the arguments you made, we would also inclue the summary figures assembled for the arbitrating referee.

If you do not want this File to be published, please inform the editorial office within 2 days, if you have not done so already, otherwise the file will be published by default [contact: emboreports@embo.org]. If you do opt out, the Review Process File link will point to the following statement: "No Review Process File is available with this article, as the authors have chosen not to make the review process public in this case." Please note that the author checklist will still be published even if you opt out of the transparent process.

Thank you again for your contribution to EMBO reports and congratulations on a successful publication. Please consider us again in the future for your most exciting work.

Yours sincerely,

Bernd Pulverer

~~~~~~~~~~~~~~~~~~~~~~~~~~~~~~~~
Bernd Pulverer, Ph.D.
Chief Editor, EMBO Reports
EMBO
Meyerhofstrasse 1, D-69117 Heidelberg
Tel: +4962218891501
bernd.pulverer@embo.org
~~~~~~~~~~~~~~~~~~~~~~~~~~~~~~~~

****

THINGS TO DO NOW:

Please note that you will be contacted by Wiley Author Services to complete licensing and payment information. The required 'Page Charges Authorization Form' is available here: https://www.embopress.org/pb-assets/embo-site/er_apc.pdf

You will receive proofs by e-mail approximately 2-3 weeks after all relevant files have been sent to our Production Office; you should return your corrections within 2 days of receiving the proofs.

Please inform us if there is likely to be any difficulty in reaching you at the above address at that time. Failure to meet our deadlines may result in a delay of publication, or publication without your corrections.

All further communications concerning your paper should quote reference number EMBOR-2021-54157V3 and be addressed to

emboreports@wiley.com.

Should you be planning a Press Release on your article, please get in contact with emboreports@wiley.com as early as possible, in order to coordinate publication and release dates.

EMBO PRESS

YOU MUST COMPLETE ALL CELLS WITH A PINK BACKGROUND ➔

PLEASE NOTE THAT THIS CHECKLIST WILL BE PUBLISHED ALONGSIDE YOUR PAPER

Corresponding Author Name: Nicolas Jaé
Journal Submitted to: Embo Reports
Manuscript Number: MBOR-2021-54157

Reporting Checklist For Life Sciences Articles (Rev. June 2017)

This checklist is used to ensure good reporting standards and to improve the reproducibility of published results. These guidelines are consistent with the Principles and Guidelines for Reporting Preclinical Research issued by the NIH in 2014. Please follow the journal's authorship guidelines in preparing your manuscript.

A- Figures
1. Data
The data shown in figures should satisfy the following conditions:

- the data were obtained and processed according to the field's best practice and are presented to reflect the results of the experiments in an accurate and unbiased manner.
- figure panels include only data points, measurements or observations that can be compared to each other in a scientifically meaningful way.
- graphs include clearly labeled error bars for independent experiments and sample sizes. Unless justified, error bars should not be shown for technical replicates.
- if n< 5, the individual data points from each experiment should be plotted and any statistical test employed should be justified
- Source Data should be included to report the data underlying graphs. Please follow the guidelines set out in the author ship guidelines on Data Presentation.

2. Captions

Each figure caption should contain the following information, for each panel where they are relevant:

- a specification of the experimental system investigated (eg cell line, species name).
- the assay(s) and method(s) used to carry out the reported observations and measurements
- an explicit mention of the biological and chemical entity(ies) that are being measured.
- an explicit mention of the biological and chemical entity(ies) that are altered/varied/perturbed in a controlled manner.
- the exact sample size (n) for each experimental group/condition, given as a number, not a range;
- a description of the sample collection allowing the reader to understand whether the samples represent technical or biological replicates (including how many animals, litters, cultures, etc.).
- a statement of how many times the experiment shown was independently replicated in the laboratory.
- definitions of statistical methods and measures:
  - common tests, such as t-test (please specify whether paired vs. unpaired), simple χ2 tests, Wilcoxon and Mann-Whitney tests, can be unambiguously identified by name only, but more complex techniques should be described in the methods section;
  - are tests one-sided or two-sided?
  - are there adjustments for multiple comparisons?
  - exact statistical test results, e.g., P values = x but not P values < x;
  - definition of 'center values' as median or average;
  - definition of error bars as s.d. or s.e.m.

Any descriptions too long for the figure legend should be included in the methods section and/or with the source data.

In the pink boxes below, please ensure that the answers to the following questions are reported in the manuscript itself. Every question should be answered. If the question is not relevant to your research, please write NA (non applicable). We encourage you to include a specific subsection in the methods section for statistics, reagents, animal models and human subjects.

USEFUL LINKS FOR COMPLETING THIS FORM

http://www.antibodypedia.com
http://1degreebio.org
http://www.equator-network.org/reporting-guidelines/improving-bioscience-research-repor
http://grants.nih.gov/grants/olaw/olaw.htm
http://www.mrc.ac.uk/Ourresearch/Ethicsresearchguidance/Useofanimals/index.htm
http://ClinicalTrials.gov
http://www.consort-statement.org
http://www.consort-statement.org/checklists/view/32-consort/66-title
http://www.equator-network.org/reporting-guidelines/reporting-recommendations-for-tumo
http://datadryad.org
http://figshare.com
http://www.ncbi.nlm.nih.gov/gap
http://www.ebi.ac.uk/ega
http://biomodels.net/
http://biomodels.net/miriam/
http://jjj.biochem.sun.ac.za
https://osp.od.nih.gov/biosafety-biosecurity-and-emerging-biotechnology/
http://www.selectagents.gov/

B- Statistics and general methods

Please fill out these boxes ➔ (Do not worry if you cannot see all your text once you press return)

1.a. How was the sample size chosen to ensure adequate power to detect a pre-specified effect size?	No statistical method was used to predetermine sample size. The sample sizes were determined according to standards and our experience in the field.
1.b. For animal studies, include a statement about sample size estimate even if no statistical methods were used.	No statistical method was used to predetermine sample size. The sample sizes were determined according to standards and our experience in the field.
2. Describe inclusion/exclusion criteria if samples or animals were excluded from the analysis. Were the criteria pre-established?	No data were excluded from the analyses.
3. Were any steps taken to minimize the effects of subjective bias when allocating animals/samples to treatment (e.g. randomization procedure)? If yes, please describe.	Randomization was not performed for cell culture experiments. Multiple, independent experiments were performed by different researchers to validate reproducibility when possible. All attempts at replication were successful.
For animal studies, include a statement about randomization even if no randomization was used.	Control mice were of the same age and experimental and control cages were randomly assigned.
4.a. Were any steps taken to minimize the effects of subjective bias during group allocation or/and when assessing results (e.g. blinding of the investigator)? If yes please describe.	The investigators were blinded during experiments and outcome assessment. These include the ECIS experiments (Figure 3 G, H, and J), in vitro permeability assays (Figures 1E, Figure EV3 K, M), in vitro sprouting assay (Figure 1F), sucrose gradients (Figure 2B, and Figure EV2E), mass specetrometry (Figure 2C), in vitro anti-TJP1 immunostaining (Figure EV3I)
4.b. For animal studies, include a statement about blinding even if no blinding was done	Invesitigator was blinded for the imaging and analysis of the vascular permeability in vivo (Figure 1H, 4G, Figure EV1M), survival and HLI ischemia model (Figure 1I), mouse retinas vascularization (Figure 4F), immune cells infiltration (Figure 4H) and H&E staining (Figure 4I).
5. For every figure, are statistical tests justified as appropriate?	Statistical significance was assessed by two-tailed paired t-test, two-tailed unpaired t-test, or Mann–Whitney U test (for non-paramatric data). Multiple comparisons were performed using one-way or two-way Anova using Tukey's, or Dunnett's correction. Probability values of less than 0.05 were considered significant. n refers to the number of independent biological replicates.
Do the data meet the assumptions of the tests (e.g., normal distribution)? Describe any methods used to assess it.	Normality and lognormality was tested using Shapiro-Wilk test.
Is there an estimate of variation within each group of data?	No

Is the variance similar between the groups that are being statistically compared?	No

C- Reagents

6. To show that antibodies were profiled for use in the system under study (assay and species), provide a citation, catalog number and/or clone number, supplementary information or reference to an antibody validation profile. e.g., Antibodypedia (see link list at top right), 1DegreeBio (see link list at top right).	anti-hnRNPL (4D11) mAb (abcam, #ab6106); GAPDH (14C10) Rabbit mAb (Cell Signalling, #2118); V450 anti-BrdU (clone 3D4 (RUO)) mAb (BD biosciences); serotype IgG control mAb (Millipore, CS200621); anti-TJP1 rAb (Thermofisher, #40-2200); biotinylated isolectin B4 (B-1205, Vector Laboratories; anti-CD45 antibodies (30F-11, BD Pharmingen)
7. Identify the source of cell lines and report if they were recently authenticated (e.g., by STR profiling) and tested for mycoplasma contamination.	HUVEC were purchased from Lonza, Lenti-X 293T Cells were acquired from Takara Biotech, HeLa cells were purchased from ATCC, H5V cells were provided by Prof. Elisabetta Dejana and Dr. Costanza Giampietro (Biomolecular Sciences and Biotechnologies, School of Sciences, University of Milan), CMT93 cells were provided by Prof. Dr. Britta Siegmund (Charite, University of Berlin). Cell lines were not authenticated. All cell lines tested negative for mycoplasma contamination. No commonly misidentified cell lines were used in this study.

* for all hyperlinks, please see the table at the top right of the document

D- Animal Models

8. Report species, strain, gender, age of animals and genetic modification status where applicable. Please detail housing and husbandry conditions and the source of animals.	For the generation of NtrasΔCA/ΔCA mice, one-cell fertelized zygotes were harvested from C57BL/6J females and electroporated with targeting sgRNAs-Cas9-complex. Viable zygotes were then transferred to CD1 pseudopregnant females. All adult mice used in this study were 12-14 weeks of age, this includes in vivo permeability assays, survival, and hindlimb ischemia (HLI) experiments. For Ntras silencing in vivo, wild type C57BL6 male and female mice were injected with LNA GapmeRs. For the HLI model only females were used. Postnatal P7 pups were used for retina assessment. As for mouse housing and husbandry conditions; mice were kept in IVC cages and living space was enriched. Mouse room and cage's temerature was kept at 20-24 degrees, humidity between 40-65%, with 12 hours of light dark cycles. Mice were always kept in same-gender social groups unless they were set for mating.
9. For experiments involving live vertebrates, include a statement of compliance with ethical regulations and identify the committee(s) approving the experiments.	All animal experiments were carried out in accordance with the principles of laboratory animal care as well as according to the German national laws. The study have been approved by the local ethic committee (Regierungspräsidium Darmstadt, Hessen)
10. We recommend consulting the ARRIVE guidelines (see link list at top right) (PLoS Biol. 8(6), e1000412, 2010) to ensure that other relevant aspects of animal studies are adequately reported. See author guidelines, under 'Reporting Guidelines'. See also: NIH (see link list at top right) and MRC (see link list at top right) recommendations. Please confirm compliance.	Complies

E- Human Subjects

11. Identify the committee(s) approving the study protocol.	NA
12. Include a statement confirming that informed consent was obtained from all subjects and that the experiments conformed to the principles set out in the WMA Declaration of Helsinki and the Department of Health and Human Services Belmont Report.	NA
13. For publication of patient photos, include a statement confirming that consent to publish was obtained.	NA
14. Report any restrictions on the availability (and/or on the use) of human data or samples.	NA
15. Report the clinical trial registration number (at ClinicalTrials.gov or equivalent), where applicable.	NA
16. For phase II and III randomized controlled trials, please refer to the CONSORT flow diagram (see link list at top right) and submit the CONSORT checklist (see link list at top right) with your submission. See author guidelines, under 'Reporting Guidelines'. Please confirm you have submitted this list.	NA
17. For tumor marker prognostic studies, we recommend that you follow the REMARK reporting guidelines (see link list at top right). See author guidelines, under 'Reporting Guidelines'. Please confirm you have followed these guidelines.	NA

F- Data Accessibility

18: Provide a "Data Availability" section at the end of the Materials & Methods, listing the accession codes for data generated in this study and deposited in a public database (e.g. RNA-Seq data: Gene Expression Omnibus GSE39462, Proteomics data: PRIDE PXD000208 etc.) Please refer to our author guidelines for 'Data Deposition'.<br><br>Data deposition in a public repository is mandatory for:<br>a. Protein, DNA and RNA sequences<br>b. Macromolecular structures<br>c. Crystallographic data for small molecules<br>d. Functional genomics data<br>e. Proteomics and molecular interactions	Done. RNA sequencing data accession number (E-MTAB-11311). Mass spectrometry data can be accessed via the accession number PXD030620.
19. Deposition is strongly recommended for any datasets that are central and integral to the study; please consider the journal's data policy. If no structured public repository exists for a given data type, we encourage the provision of datasets in the manuscript as a Supplementary Document (see author guidelines under 'Expanded View' or in unstructured repositories such as Dryad (see link list at top right) or Figshare (see link list at top right).	Datasets are provided as tables EV1-3, mass spectrometry data (table EV1), RNA-sequencing data (tables EV2 and EV3).
20. Access to human clinical and genomic datasets should be provided with as few restrictions as possible while respecting ethical obligations to the patients and relevant medical and legal issues. If practically possible and compatible with the individual consent agreement used in the study, such data should be deposited in one of the major public access-controlled repositories such as dbGAP (see link list at top right) or EGA (see link list at top right).	NA
21. Computational models that are central and integral to a study should be shared without restrictions and provided in a machine-readable form. The relevant accession numbers or links should be provided. When possible, standardized format (SBML, CellML) should be used instead of scripts (e.g. MATLAB). Authors are strongly encouraged to follow the MIRIAM guidelines (see link list at top right) and deposit their model in a public database such as Biomodels (see link list at top right) or JWS Online (see link list at top right). If computer source code is provided with the paper, it should be deposited in a public repository or included in supplementary information.	NA

G- Dual use research of concern

22. Could your study fall under dual use research restrictions? Please check biosecurity documents (see link list at top right) and list of select agents and toxins (APHIS/CDC) (see link list at top right). According to our biosecurity guidelines, provide a statement only if it could.	NA
